# The Histone Lysine Demethylase KDM7A Contributes to Reward Memory via Fscn1‐Induced Synaptic Plasticity in the Medial Prefrontal Cortex

**DOI:** 10.1002/advs.202405352

**Published:** 2025-01-21

**Authors:** Zhuo‐jin Yang, Dong‐yu Yu, Fei‐fei Gao, Dan‐ya Zhou, Ya‐nan Wu, Xi‐xi Yang, Jie Chen, Jing‐si Yang, Meng‐qing Shen, Yu‐xiang Zhang, Lai Wei, Chun‐xia Yan

**Affiliations:** ^1^ College of Forensic Medicine Key Laboratory of National Health Commission for Forensic Medicine Xi'an Jiaotong University Health Science Center Xi'an Shaanxi 710061 China; ^2^ Xinxiang Key Laboratory of Forensic Toxicology School of Forensic Medicine Xinxiang Medical University Xinxiang Henan 453003 China

**Keywords:** Fscn1, histone demethylase KDM7A, medial prefrontal cortex, morphine, reward memory

## Abstract

Lysine demethylase 7A (KDM7A) catalyzes the removal of dimethylation from histone H3 lysine 9 and lysine 27, both of which are associated with transcription repression. Previous study indicates that Kdm7a mRNA in the medial prefrontal cortex (mPFC) increases after drug exposure, yet its role in drug‐related behaviors is largely unknown. In a morphine‐conditioned place preference (CPP) paradigm, these findings reveal a specific increase of Kdm7a expression in the mPFC 7 days after drug withdrawal. Subsequently, these results demonstrate that knockdown of Kdm7a in the mPFC do not affect the acquisition of morphine‐induced CPP, but it attenuate memory consolidation. To further explore Kdm7a‐mediated transcriptomic changes, this work employs Nanopore direct RNA sequencing. Transcriptome profiling unveils several gene expression alterations impacted by KDM7A, which are enriched in relevant neural function categories. Notably, this work identifies and validates fascin actin‐bundling protein 1 (Fscn1) as a downstream molecular target. Knockdown of Fscn1 has a similar impact on CPP to Kdm7a, along with corresponding decrease of dendritic spine density and neuronal activity in the mPFC. Additionally, silencing Kdm7a decreases enrichment of H3K9me2 and H3K27me2 at the Fscn1 promoter region, suggesting that KDM7A may act as a crucial regulator of transcriptional responses to morphine‐related reward memory via Fscn1.

## Graphical abstract

This research delineates the role of lysine demethylase 7A (KDM7A) in morphine‐induced reward memory and its effects on gene expression in the medial prefrontal cortex (mPFC). The study identifies fascin actin‐bundling protein 1 (Fscn1) as a downstream target of KDM7A, affecting dendritic spine density and neuronal activity, revealing novel insights into the molecular underpinnings of addiction.

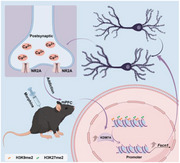

## 1 Introduction

Drug addiction is a chronic and relapsing brain disorder.^[^ ^1^ ^]^ Mounting evidence has shown that epigenetic mechanisms play a significant role in drug addiction and relapse.^[^ ^2^ ^]^ Epigenetics is to remodel gene expression without changing the DNA sequence, and epigenetic regulatory events include histone post‐translational modifications, DNA modifications and the actions of non‐coding RNAs.^[^ ^3^ ^]^ The N‐terminal tail of histones are the main sites of post‐translational modifications, which can be modified by acetylation, methylation, phosphorylation, ubiquitination and SUMOylation.^[^ ^4^ ^]^ The most studied histone modifications in drug addiction are histone acetylation and histone methylation.^[^ ^5^, ^6^, ^7^, ^8^, ^9^ ^]^ Histone methylation could alter chromatin structure and the accessibility of DNA to transcriptional machinery. This occurs by virtue of the loosening or binding of histone tails around the DNA, thereby either permitting or restricting access to cell‐specific transcription factors, initiation factors, elongation factors, and other associated proteins.^[^ ^10^ ^]^ Epigenetics plays a pivotal role in drug‐induced dynamic changes of synaptic plasticity, including both structural and functional plasticity.^[^ ^11^ ^]^ For example, actin, a key component of the cytoskeleton, plays a significant role in the cellular mechanisms underlying drug addiction. Actin dynamics are crucial for the morphological changes that occur in neurons in response to addictive drugs. The reorganization of the actin cytoskeleton facilitates the formation of new synaptic connections and the strengthening of existing ones, which are hallmarks of drug‐induced neuroplasticity. These changes contribute to the encoding and retention of drug‐associated memories, which play a significant role in addiction‐related behaviors.^[^ ^12^, ^13^ ^]^ Unlike DNA sequence changes, many epigenetic modifications are reversible, which may provide broad prospects for the treatment of various diseases.^[^ ^14^ ^]^ Therefore, in‐depth investigation of the epigenetic mechanisms involved in the formation and maintenance of drug addiction‐related memories will contribute to the prevention and treatment of clinical drug addiction and relapse.

Post‐transcriptional modifications of histone tails play a crucial role in either activating or repressing the transcription of target genes, depending on specific residues.^[^ ^10^ ^]^ The Jumonji C domain‐containing enzymes comprise one of the most common family of histone demethylases (KDMs),^[^ ^14^ ^]^ which could specifically demethylase lysine residues. Among these, KDM7A, also known as JHDM1D or KIAA1718, contains a jumonji domain responsible for H3K9me2 and H3K27me2.^[^ ^15^ ^]^ H3K9me2 and H3K27me2 are primarily associated with gene repression.^[^ ^16^ ^]^

Previous research on KDM7A has predominantly focused on its involvement in brain development, cell differentiation, cell cycle, cell proliferation, inflammatory responses of endothelial cells, and various kinds of cancer.^[^ ^15^, ^17^, ^18^ ^]^ In these contexts, KDM7A functions as an eraser of repressive marks on chromatin. Initial transcriptomic findings from animal models have indicated a notable upregulation of Kdm7a gene expression in the prefrontal cortex following exposure to drugs.^[^ ^19^ ^]^ However, the precise role and underlying mechanisms of Kdm7a in addiction remain to be fully elucidated.

FSCN1 is a highly conserved actin‐binding protein that interacts with filamentous actin and facilitates the formation of tight bundles through its actin‐binding domain.^[^ ^20^, ^21^ ^]^ This molecular mechanism is crucial for fundamental processes such as cell movement and the maintenance of cellular morphology. Notably, during embryogenesis, FSCN1 demonstrates broad expression in developing neural systems and mesenchymal tissues.^[^ ^22^ ^]^ Furthermore, FSCN1 has been implicated in various cellular functions, particularly in processes associated with protrusion, including filopodia and lamellipodia formation.^[^ ^23^, ^24^ ^]^

In this study, first we assessed both RNA and protein expression of Kdm7a in addiction‐associated brain regions. We found Kdm7a mRNA exhibited a specific increase in the mPFC at 3 and 7 days post‐withdrawal, while no significant alteration was observed in the nucleus accumbens (NAc), caudate putamen (CPu), and hippocampus. Consistently, Western blotting analysis indicated an elevation of KDM7A protein levels at 3 days post‐withdrawal, peaking at 7 days and returning to baseline by 30 days. These findings suggested spatial and temporal expression of KDM7A. Besides, Kdm7a knockdown in the mPFC did not impact morphine‐induced CPP acquisition but attenuated CPP consolidation. Transcriptome profiling unveiled numerous Kdm7a‐mediated gene expression alterations, which were enriched in relevant neuronal function categories. Intriguingly, these Kdm7a‐regulated gene expression changes displayed substantial overlap with morphine‐induced transcriptomic modifications. Further qRT‐PCR validation revealed Fscn1 as a potential downstream target of KDM7A. Fscn1 knockdown in the mPFC significantly impaired CPP consolidation, indicating its inhibitory effect on morphine‐related reward memory. Moreover, ChIP‐qPCR experiments confirmed the regulatory role of H3K9me2 and H3K27me2 modifications at the promoter region of Fscn1. Knockdown of Kdm7a or Fscn1 in the mPFC reversed morphine‐induced elevation of neuronal activity and dendritic spine density of prefrontal neurons. In summary, our findings delineated that KDM7A in the mPFC may function as an endogenous molecular modulator of morphine‐related reward memory through Fscn1.

## 2 Results

### 2.1 Significant Upregulation of KDM7A Expression in the mPFC After Morphine Withdrawal

**Figure 1:**
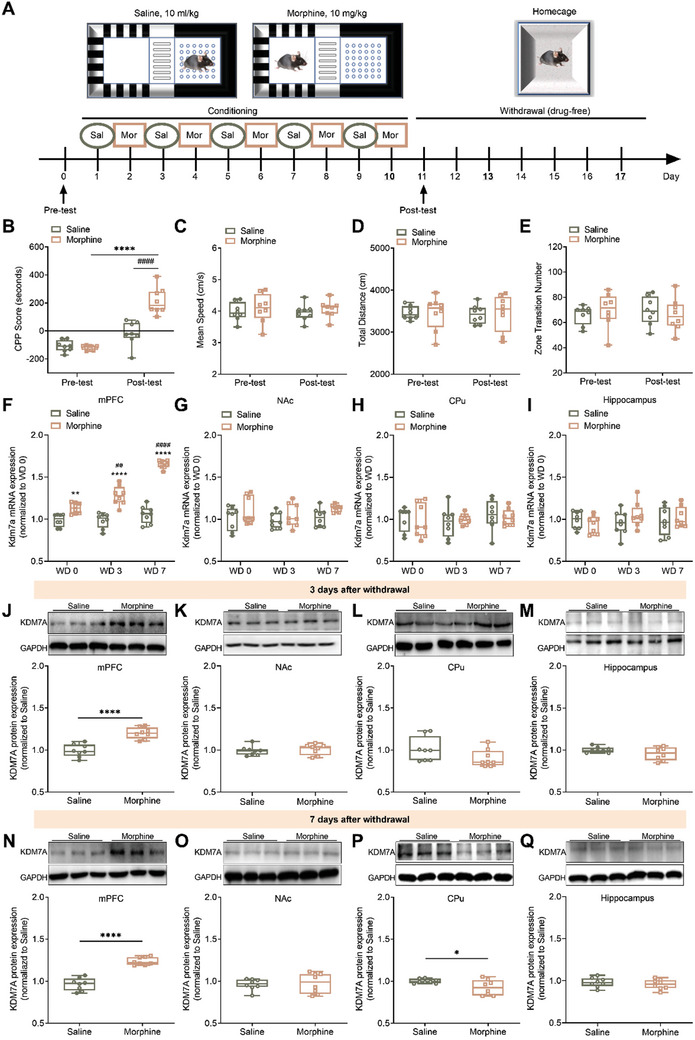
The spatial and temporal up‐regulation of KDM7A after morphine withdrawal. A) The timeline of CPP paradigm: after 10‐day conditioning, post‐test was performed on Day 11, and mPFC tissue was extracted on Day 10, 13, 17 (WD 0, WD 3, WD 7). B) CPP score between saline and morphine groups at post‐test (n = 8). The data were presented as the mean ± SEM; Two‐way ANOVA and Bonferroni's multiple comparisons test. ^####^ *p* < 0.0001, ****p < 0.0001. C‐E) There was no significant difference of other locomotion parameters between saline and morphine groups during pre‐test and post‐test (n = 8). The data were presented as mean ± SEM. Two‐way ANOVA and Bonferroni's multiple comparisons test. C) Mean speed (cm s^−1^). D) Total distance (cm) during 15 min activity. E) Zone transition number during 15 min. F–I) Morphine‐induced changes of Kdm7a mRNA expression in the mPFC, NAc, CPu and hippocampus during different withdrawal stages compared to saline group (n = 8). The data were presented as the mean ± SEM; Two‐Way ANOVA and Bonferroni's multiple comparisons test. **p < 0.01, ****p < 0.0001(compared to their corresponding saline group). ^##^ *p* < 0.01, ^####^ *p* < 0.0001(compared to morphine group at WD 0). J–M) Changes of KDM7A protein expression in the mPFC, NAc, CPu and hippocampus on Day 13 (n = 8). The data were presented as the mean ± SEM; Unpaired *t*‐test; ****p < 0.0001. N‐Q) Changes of KDM7A protein expression in the mPFC, NAc, CPu and hippocampus on Day 17 (n = 8). The data were presented as the mean ± SEM; Unpaired *t*‐test. *p < 0.05, ****p < 0.0001.

Mice received alternate saline and morphine (10 mg kg^−1^) administration for 10 days (**Figure** **1**A). CPP score was calculated by subtracting time in non‐drug chamber from drug chamber. In the post‐test, the CPP score of the morphine group was significantly higher than that of the saline group, and there was also a significant difference compared to the CPP score in the pre‐test. The results indicated the successful establishment of morphine‐induced CPP (Figure 1B). Additionally, other locomotor parameters, including mean speed, total distance, and zone transition number, were assessed (Figure 1C–E). Notably, neither the drug treatment (saline versus morphine) nor the testing time (pre‐test versus post‐test) had any significant effect on locomotor activity, excluding that the drug may cause any effect on basal locomotion.

The mRNA expression levels of Kdm7a in the mPFC, NAc, CPu, and hippocampus of each group were evaluated using the qRT‐PCR. Figure 1F demonstrated that the Kdm7a mRNA in the mPFC increased significantly at 0, 3, and 7 days after morphine withdrawal. In Figure 1G, no significant difference was observed among the four groups in the NAc. Similarly, no significant difference of Kdm7a mRNA expression was found in either the CPu or the hippocampus between the two groups (Figure 1H,I).

We also quantified the protein expression of KDM7A in the mPFC, NAc, CPu, and hippocampus. Figure 1J–M indicated the protein expression of KDM7A between saline and morphine groups after 3 days of withdrawal. The morphine group exhibited significantly higher expression of KDM7A compared to the saline group in the mPFC. However, no significant difference was observed of KDM7A protein expression in the NAc, CPu, and hippocampus. These findings suggested an elevated expression of KDM7A protein in the mPFC after 3 days of withdrawal.

Similarly, KDM7A protein expression in the mPFC, NAc, CPu, and hippocampus was also examined after 7 days of withdrawal (Figure 1N–Q). The results indicated that following 7 days of withdrawal, there was a significant increase of KDM7A expression in the mPFC. No significant difference of KDM7A protein was observed in either the NAc or the hippocampus between the two groups. However, in the CPu, the expression of KDM7A decreased in comparison to the saline group.

Furthermore, the expression of KDM7A protein in the mPFC was examined after 30 days of withdrawal (Figure S1, Supporting Information). Our results showed no significant difference of KDM7A protein expression in the mPFC after 30 days drug‐free, suggesting that KDM7A protein expression had recovered to the saline level.

### 2.2 Kdm7a Knockdown Attenuated Memory Consolidation

**Figure 2:**
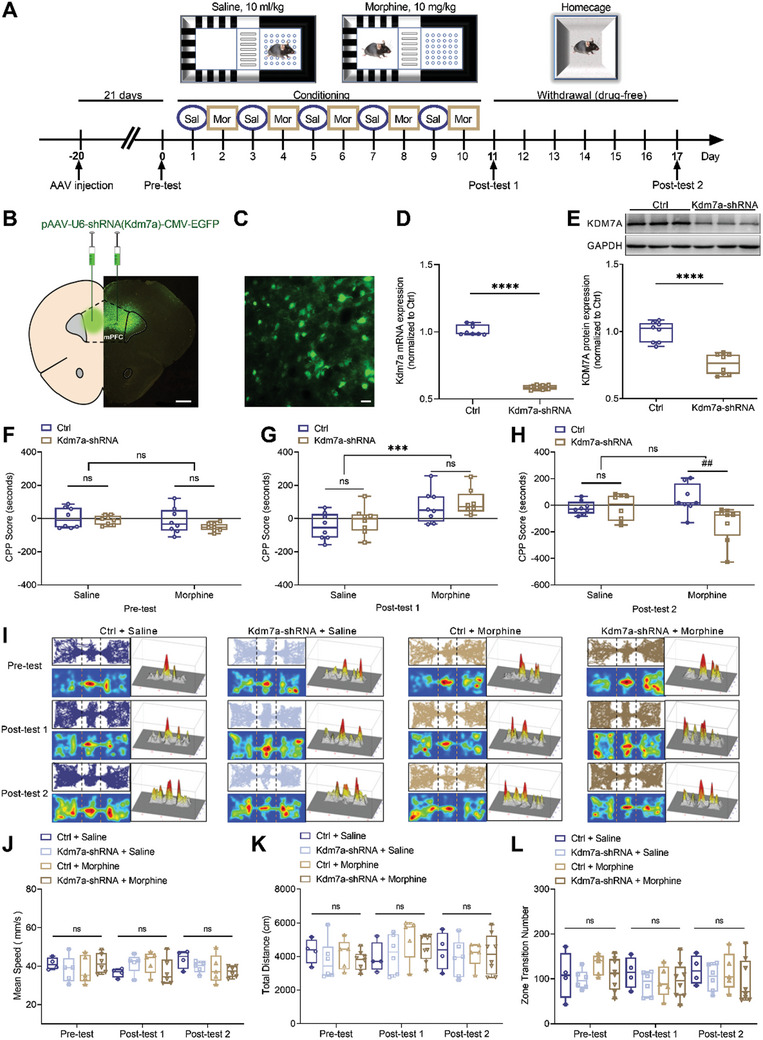
Kdm7a knockdown attenuated memory consolidation. A) The timeline of CPP paradigm: Kdm7a‐shRNA virus was injected 20 days before pre‐test; after 10 days of conditioning, mice received two post‐tests on Day 11 and Day 17, and mPFC tissue was extracted after post‐test on Day 17. B) Virus localization and expression in the mPFC. Bar = 500 µm. C) Representative figure of AAV‐ shRNA (Kdm7a)‐ EGFP in mPFC. Bar = 40 µm. D) Kdm7a mRNA expression in the mPFC decreased after Kdm7a knockdown (n = 8). The data were presented as the mean ± SEM; Unpaired *t*‐test; ****p < 0.0001. E) Significant decrease of KDM7A protein expression in Kdm7a knockdown virus group compared to Ctrl virus group (n = 8). The data were presented as the mean ± SEM; Unpaired t test; ****p < 0.0001. F) CPP score of pre‐test (n = 8). The data were presented as the mean ± SEM; Two‐way ANOVA and Bonferroni's multiple comparisons test. G) CPP score of post‐test 1 after the last injection of morphine (n = 8). The data were presented as the mean ± SEM; Two‐way ANOVA and Bonferroni's multiple comparisons test. ***p < 0.001. H) CPP score of post‐test 2 on Day 17 (n = 8). The data were presented as the mean ± SEM; Two‐way ANOVA and Bonferroni's multiple comparisons test. ^##^ *p* < 0.01. I) Trajectory maps, trajectory heat maps and trajectory maps in 3D of four groups during pre‐test, post‐test 1 and post‐test 2. J–L) Other behavioral parameters, such as mean speed, total distance and zone transition number were not significantly different among different groups and tests (n = 4–8). The data were presented as the mean ± SEM; Two‐way ANOVA and Bonferroni's multiple comparisons test.

To assess the role of KDM7A in morphine‐induced reward memory, we knocked down Kdm7a by microinjecting pAAV‐U6‐shRNA (Kdm7a)‐CMV‐EGFP‐WPRE into the mPFC (**Figure** **2**A–C). After 10 days of conditioning, mice received two post‐tests on Day 11 and Day 17.

First, we examined both the mRNA and protein expression to measure the knockdown efficiency, and we found both the mRNA and protein of Kdm7a decreased after Kdm7a‐shRNA virus injection (Figure 2D,E). Kdm7a knockdown had no effect on the post‐test 1 (Figure 2G), suggesting that KDM7A was not required for memory formation. However, Kdm7a knockdown significantly attenuated morphine‐induced reward memory during the post‐test 2, while the knockdown itself had no effect on the CPP score (Figure 2H,I). These findings indicated that KDM7A activity was required for memory consolidation. The locomotion activity was also measured, and there was no difference between scramble and knockdown groups (Figure 2J–L), excluding the possibility that Kdm7a knockdown had an impact on basal locomotion.

To investigate whether the effect of KDM7A on memory is specific to reward memory, we injected Kdm7a‐shRNA or NC‐shRNA into the mPFC, and Morris water maze (MWM) was performed to evaluate spatial learning and memory. Briefly, mice received 5 days of learning trials, followed by two probe test (Figure S2A, Supporting Information). Our results showed that Kdm7a knockdown had no effect on swimming speed, excluding any potential side effects on motor performance (Figure S2B, Supporting Information). During the learning phase, as the mice underwent more training trials, the escape latency decreased. However, there was no significant difference between the two groups (Figure S2C, Supporting Information). During the probe test, there was no significant difference between the two groups regarding the number of platform site crossings (Figure S2D, Supporting Information), the time in the target quadrant (Figure S2E, Supporting Information), and the time in original platform location (Figure S2F, Supporting Information). These findings indicated that KDM7A activity was not required for spatial memory.

### 2.3 KDM7A is a Key Regulatory Factor in Morphine‐Induced Transcriptional Changes

**Figure 3:**
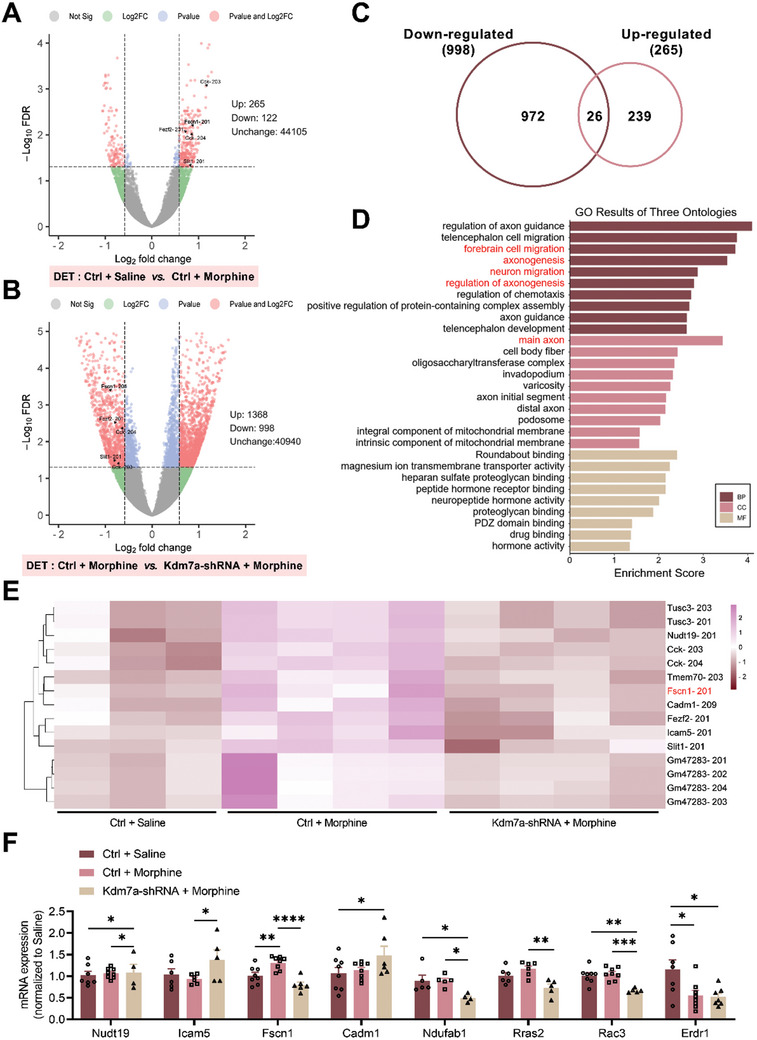
Transcriptome analysis and downstream target gene screening of KDM7A. A) Volcano plot indicating the DET of Ctrl + Saline versus Ctrl + Morphine group. Fold Change > 1.5. B) Volcano plot indicating the DET of Ctrl + Morphine versus Kdm7a‐shRNA + Morphine group. C) Intersection of 26 differentially expressed transcripts that were downregulated in Ctrl + Morphine versus Kdm7a‐shRNA + Morphine and upregulated in Ctrl + Saline versus Ctrl + Morphine group. D) GO annotation and enrichment analysis of differentially expressed transcripts using standard bioinformatics analysis methods, only terms closely related to development of neural system were shown. E) Heatmap showing 15 DET. Pink gradient: positive fold change and red gradient: negative fold change. F) qRT‐PCR validation of 8 representative differentially expressed transcripts (n = 4–8). The data were presented as the mean ± SEM; Two‐way ANOVA and Bonferroni's multiple comparisons test. *p < 0.05, **p < 0.01, ***p < 0.001, ****p < 0.0001.

KDM7A is a histone lysine demethylase that removes methyl groups from lysine 9 and lysine 27 of histone H3, resulting in activation of target genes. In order to identify KDM7A targets, the mPFC tissue was dissected immediately after post‐test 2, and third‐generation Nanopore sequencing was conducted. A total of 8564 differential expressed transcripts (DET) were identified (Table S1, Supporting Information). Among these transcripts, there were 387 differential transcripts between the Ctrl + Saline and Ctrl + Morphine groups (265 upregulated and 122 downregulated) (**Figure** **3**A). Additionally, there were 2366 differential transcripts between the Ctrl + Morphine and Kdm7a‐shRNA + Morphine groups (1368 upregulated and 998 downregulated) (Figure 3B). As shown in Table S1, Supporting Information, there were 5754 differential transcripts between the Ctrl + Saline and Kdm7a‐shRNA + Saline groups (3140 upregulated and 2614 downregulated), and 57 differential transcripts between the Kdm7a‐shRNA + Saline and Kdm7a‐shRNA + Morphine groups (19 upregulated and 38 downregulated). The intersection of downregulated transcripts in the Ctrl + Morphine versus Kdm7a‐shRNA + Morphine groups and upregulated transcripts in the Ctrl + Saline versus Ctrl + Morphine groups yielded 26 differential transcripts (Figure 3C).

As depicted in Figure 3D, standard bioinformatics analysis including Gene Ontology (GO) annotation and enrichment analysis was conducted on all differential transcripts. In the biological process (BP) category, the differential transcripts were primarily involved in the regulation of axon guidance, telencephalon cell migration, forebrain cell migration, and neuron migration. In the cellular component (CC) category, the differential transcripts were mainly enriched in the main axon, cell body fiber, and oligosacchary ltransferase complex. In the molecular function (MF) category, these differential transcripts were enriched in binding, transporter activity, and neuropeptide hormone activity. The heatmap in Figure 3E showed the hierarchical clustering analysis of the top 15 transcripts.

To validate the accuracy of the sequencing results, we conducted qRT‐PCR using mPFC tissue collected on the seventh day of morphine withdrawal in mice. The differentially expressed genes which have been previously reported to be associated with drug addiction were selected. Among these candidates, Fscn1 showed high consistency with the sequencing data. Specifically, Fscn1 mRNA expression was significantly elevated after morphine withdrawal, whereas decreased after Kdm7a knockdown, suggesting that Fscn1 may serve as a downstream target gene of KDM7A (Figure 3F).

To further confirm that Fscn1 is a downstream target gene of KDM7A, we assessed the mRNA and protein expression of Kdm7a in the mPFC after Fscn1 knockdown. The results showed that there was no difference between Fscn1‐shRNA and Ctrl groups, indicating that knockdown of Fscn1 had no effect on the expression of KDM7A (Figure S3, Supporting Information).

### 2.4 Fscn1 Knockdown Attenuated Memory Consolidation

**Figure 4:**
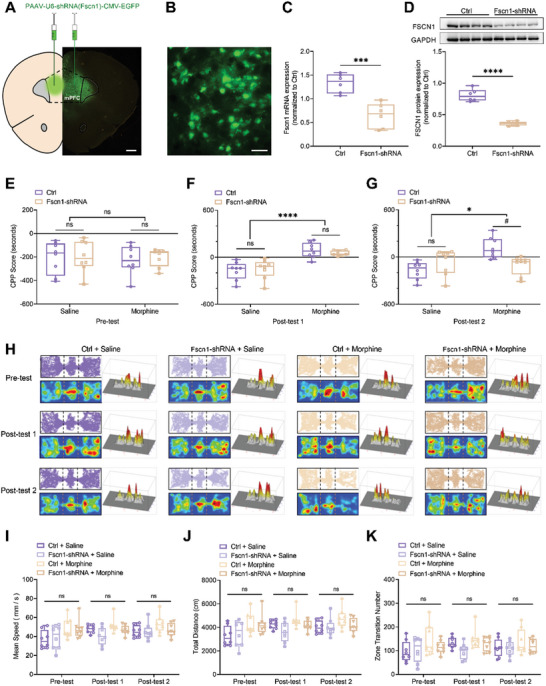
Inhibition of morphine addiction memory after Fscn1 interference. A) Schematic diagram of microinjection into mPFC and brain atlas of mPFC. Bar = 500 µm. B) Fluorescent localization of mPFC in mouse. Bar = 40 µm. C,D) Decrease of Fscn1 mRNA and protein expression in mPFC of mice after Fscn1 knockdown (n = 6). The data were presented as the mean ± SEM; Unpaired t test; ***p < 0.001, ****p < 0.0001. E) CPP score of pre‐test (n = 8). The data were presented as the mean ± SEM; Two‐way ANOVA and Bonferroni's multiple comparisons test. F) CPP score of post‐test 1 (n = 8). The data were presented as the mean ± SEM; Two‐way ANOVA and Bonferroni's multiple comparisons test. ****p < 0.0001. G) CPP score of post‐test 2 (n = 8). The data were presented as the mean ± SEM; Two‐way ANOVA and Bonferroni's multiple comparisons test. *p < 0.05. ^#^ *p* < 0.05. H) Trajectory maps, trajectory heat maps and trajectory maps in 3D of Ctrl + Saline and Fscn1‐shRNA + Saline or Ctrl + Morphine and Fscn1‐shRNA + Morphine group during the pre‐test, post‐test 1 and post‐test 2. I–K) Other behavioral parameters, such as mean speed, total distance and zone transition number were not significantly different among different groups and test (n = 8). The data were presented as the mean ± SEM; Two‐way ANOVA and Bonferroni's multiple comparisons test.

Next, we tried to decipher the function of Fscn1 in morphine‐induced reward memory. We microinjected pAAV‐U6‐shRNA (Fscn1)‐CMV‐EGFP‐WPRE into the mPFC (**Figure** **4**A,B). After the behavioral experiment on Day 17, the mPFC tissue was extracted to verify virus injection and expression. The results shown in Figure 4C demonstrated that the mRNA of Fscn1 in the knockdown group was decreased, consistent with the decrease of FSCN1 protein expression (Figure 4D), further validating the efficiency of the virus.

Fscn1 knockdown had no effect on the post‐test 1 (Figure 4F), suggesting that Fscn1 was not required for memory formation. However, Fscn1 knockdown significantly attenuated the conditioned memory during the post‐test 2 (Figure 4G,H). These findings indicated that Fscn1 activity was required for conditioned memory consolidation.

We also examined the potential effects of Fscn1 knockdown on basal locomotor activity, mean speed and zone transition number, and found no significant effect during post‐test 2 (Figure 4I–K). These results corroborated our findings in Figure 3F, further demonstrating that Fscn1 knockdown is able to attenuate memory strength during consolidation without influencing exploration in the test apparatus.

To elucidate the combined role of KDM7A and FSCN1 in morphine reward memory, we also constructed pAAV‐hSyn‐Kdm7a‐3xFLAG‐WPRE and pAAV‐hSyn‐mCherry‐P2A‐Fscn1‐3xFLAG‐WPRE to overexpress Kdm7a and Fscn1, respectively. The results showed that Kdm7a overexpression had no effect on morphine‐induced CPP score, compared with corresponding Ctrl group. Besides, double knockdown significantly attenuated morphine‐induced reward memory during the post‐test 2, which is similar with the effect of single Kdm7a or Fscn1 knockdown. However, overexpression of Fscn1 counteracted the effects of Kdm7a knockdown on morphine‐related memory consolidation (Figure S4, Supporting Information).

To further investigate the roles of KDM7A and FSCN1 in other stages of drug addiction, we conducted additional experiments during the reinstatement phase of morphine‐induced CPP in mice. After 10 consecutive days of morphine conditioning, the post‐test 1 and post‐test 2 were similarly performed as described above. On WD 24, all the mice received post‐test 3 immediately after morphine injection for reinstatement. We found that morphine priming dramatically increased CPP score, and Kdm7a or Fscn1 knockdown significantly attenuated the CPP score during post‐test 3 (Figure S5, Supporting Information). Thus, it is plausible that the morphine‐induced long‐term memory is dependent on the early consolidation.

### 2.5 Kdm7a or Fscn1 Knockdown Induced Abnormal Dendritic Morphogenesis in the mPFC

**Figure 5:**
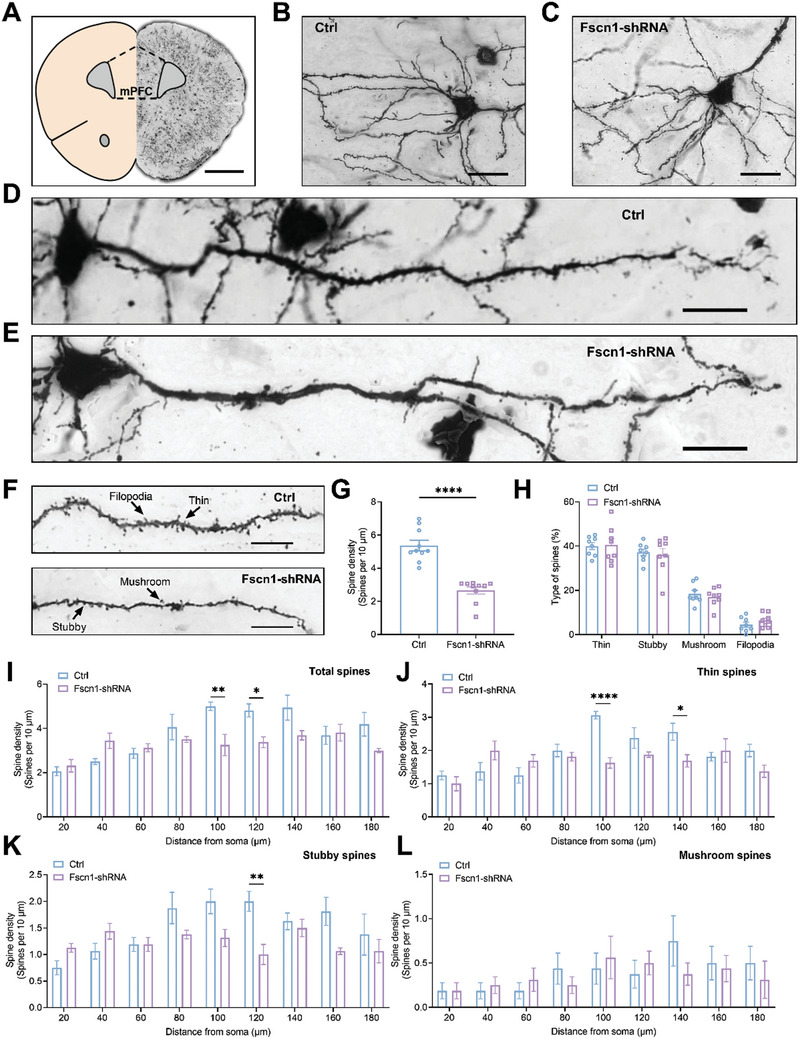
Fscn1 knockdown resulted in loss of spines in the mPFC. A) Golgi‐stained mPFC sections which were extracted after post‐test on Day 17, scale bar = 1 mm. B,C) Representative basal dendrites of Ctrl and Fscn1‐shRNA mice. Bar = 25 µm. D,E) Representative apical dendrites from Ctrl and Fscn1‐shRNA mice; thin, stubby and mushroom‐type spines were showed by arrows. Bar = 20 µm. F) The representative magnified parts of dendrites taken from Ctrl mouse and Fscn1‐shRNA mouse. Bar = 10 µm. G) A decreased spine density was apparent in Fscn1‐shRNA mice (n = 10). The data were presented as the mean ± SEM. Unpaired t test. ****p < 0.0001. H) No difference was found in thin, stubby, mushroom spine fraction between Ctrl and Fscn1‐shRNA mice (n = 8). The data were presented as the mean ± SEM. Two‐way ANOVA and Bonferroni's multiple comparisons test. I–L) Fscn1‐shRNA mice loss of spines: including both thin and stubby ‐type spines were primarily observed on apical dendrites that were 100–140 µm from the soma of pyramidal cells (n  =  8). The data were presented as the mean ± SEM. Two‐way ANOVA and Bonferroni's multiple comparisons test. *p < 0.05, **p < 0.01, ****p < 0.0001. No difference was observed on mushroom‐type spines.

As FSCN1 is indispensable for the formation of different kinds of cell protrusions, the Golgi staining and analysis were performed to assess the dendritic spine density in the mPFC (**Figure** **5**A). We compared apical dendrites from control and Fscn1‐shRNA mice (Figure 5B–F). Our results revealed a significant reduction in dendritic spine density in mPFC after Fscn1 was knocked down (Figure 5G). As reported in previous studies, spines were further classified into mushroom, stubby, thin and filopodia spines.^[^ ^25^ ^]^ Mushroom spines exemplify the prototypical dendritic spine morphology, characterized by a large head, a thin neck, a long lifespan, and remarkable stability.^[^ ^26^, ^27^, ^28^ ^]^ Thin spines possess minute heads and necks, which are highly plastic structures capable of rapid expansion into long‐lasting mushroom spines. Stubby spines are considered to be transitional structures.^[^ ^29^ ^]^ Filopodia spines are widely recognized as precursors to dendritic spines, playing a crucial role in establishing new synapses with neighboring axons.^[^ ^30^ ^]^

The percentage of thin, stubby‐type, mushroom‐type and filopodia spines was comparable between the two groups (Figure 5H). The post hoc test indicated that spine loss in the Fscn1 knockdown mice derived from a lower density of spines on apical dendritic segments at 100–140 µm from the soma (Figure 5I–L). Specifically, neuron density from Fscn1 knockdown mice had significant reduction in both thin and stubby‐type spines compared with neurons from control mice. Due to the low number of filopodia spines, no statistical analysis was conducted on them. Taken together, these data suggested that FSCN1 selectively affects spines on apical dendrites of pyramidal cells, leading to loss of both thin and stubby‐type spines in the mPFC.

Spines are major sites of excitatory synapses, thus dendritic spines form a compartment for processing the individual synaptic input. The NR2A subunit is a component of the N‐methyl‐D‐aspartate (NMDA) receptor, which is a type of ionotropic glutamate receptor in synapses. NMDA receptors, including those containing the NR2A subunit, are primarily located on the postsynaptic membrane of neurons. To further elucidate the molecular mechanisms of FSCN1 on synaptic plasticity, we microinjected Fscn1‐shRNA into the mPFC, and performed Western blotting to assess the changes of NR2A expression. On the seventh day after morphine withdrawal, mPFC brain tissue was collected. Our results showed that NR2A expression was enhanced after morphine withdrawal, and knockdown of Fscn1 reversed this morphine‐induced increase of NR2A (Figure S6, Supporting Information).

**Figure 6:**
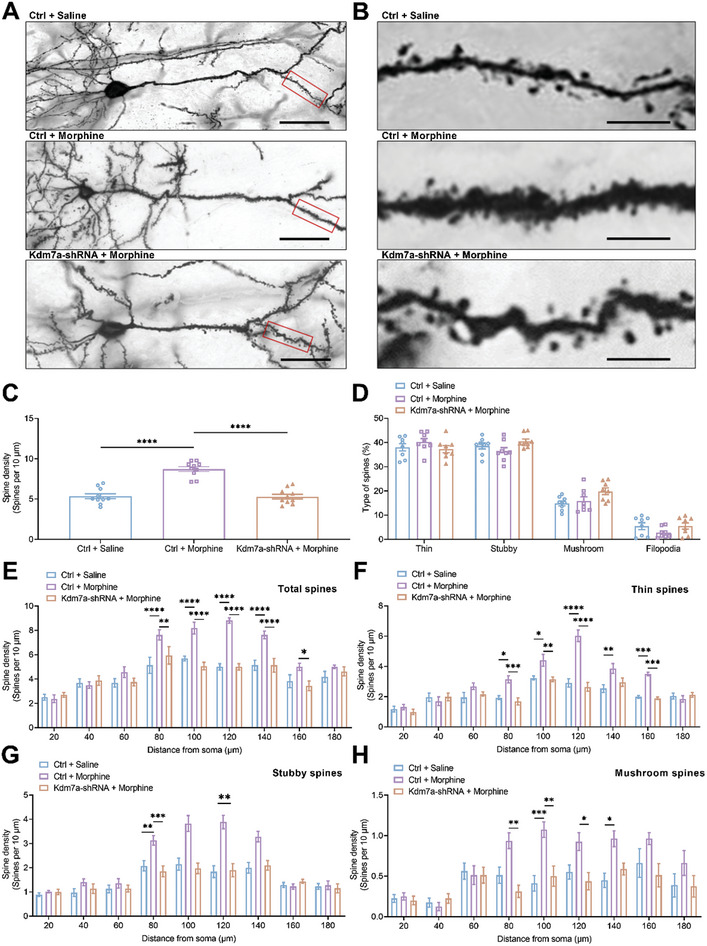
Knockdown of Kdm7a blocked morphine‐induced increase of dendritic spines. A) Representative apical dendrites of Ctrl + Saline, Ctrl + Morphine and Kdm7a‐shRNA + Morphine mice. Bar = 50 µm. B) The boxed segments from A were magnified. Bar = 10 µm. C) The spine density of Ctrl + Saline, Ctrl + Morphine and Kdm7a‐shRNA + Morphine mice (n = 10). The data were presented as the mean ± SEM; One‐way ANOVA and Bonferroni's multiple comparisons test; ****p < 0.0001. D) The percentage of thin, stubby and mushroom‐type spines was not affected (n = 8). The data were presented as the mean ± SEM; Two‐way ANOVA and Bonferroni's multiple comparisons test. E‐H) Changes of total spine density, including 3 different types of spines, were primarily observed on apical dendrites that were 80–160 µm from the soma of pyramidal cells (n = 8). The data were presented as the mean ± SEM; Two‐way ANOVA and Bonferroni's multiple comparisons test. *p < 0.05, **p < 0.01, ***p < 0.001, ****p < 0.0001.

Previous study has suggested that KDM7A plays a crucial role in synaptic plasticity and cognitive processes.^[^ ^31^ ^]^ We also conducted Golgi staining on mPFC slices immediately after the post‐test 2. Our results showed that the dendritic spine density in the Ctrl + Morphine group was significantly elevated compared to the Ctrl + Saline group, while the dendritic spine density in the Kdm7a‐shRNA + Morphine group was significantly decreased compared to the Ctrl + Morphine group (**Figure** **6**A–C). Overall, the percentage of the four types of dendritic spines was similar among three groups (Figure 6D). Specifically, the dynamic changes of dendritic spine density were mainly on the apical dendritic segments located 80–160 µm away from the soma, including thin, stubby and mushroom spines (Figure 6E–H).

Our results demonstrated that Kdm7a knockdown significantly attenuated morphine‐induced reward memory during the post‐test 2 (Figure 2H), accompanied by decreased dendritic spine density in the mPFC. Taken together, these data suggested that spine elimination in the mPFC contributed to impaired memory consolidation.

### 2.6 Downregulation of Kdm7a/ Fscn1 in the mPFC Blocked Morphine‐Induced Elevation of Neuronal Activity

**Figure 7:**
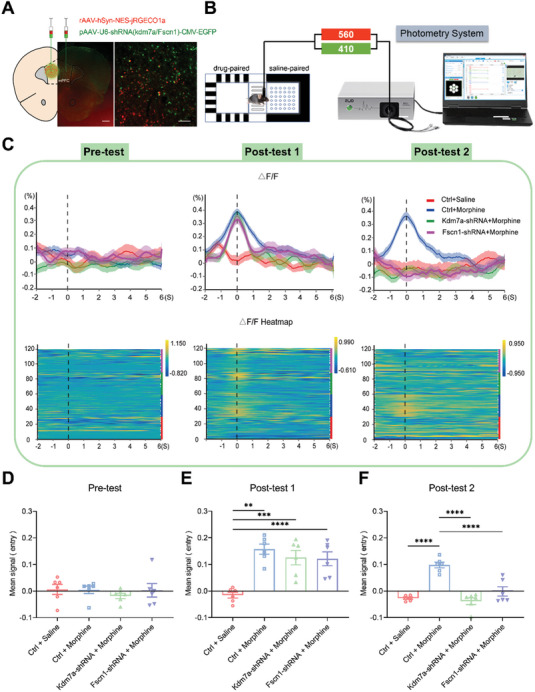
Knockdown of Kdm7a/ Fscn1 blocked morphine‐induced elevation of neuronal activity in the mPFC. A) Virus localization and expression in the mPFC, which were extracted after post‐test on Day 17. Bar1 = 500 µm. Bar2 = 80 µm. B) Diagram of fiber photometry for recording RGECO1a activity from mPFC neurons before and after morphine‐induced CPP. Calcium‐dependent (560 nm) and calcium‐independent (410 nm) fluorescence signals were recorded. C) ΔF/F of calcium signals from Ctrl + Saline group (red line), Ctrl + Morphine (blue line), Kdm7a‐shRNA + Morphine (green line) and Fscn1‐shRNA + Morphine (purple line) neurons were recorded before (2 s) and after entry into the drug‐paired chamber (6 s) during pre‐test, post‐test 1 and post‐test 2. Heatmaps illustrating Ca^2+^ signals aligned to the initiation of trials during pre‐test, post‐test 1 and post‐test 2. Each row plots one trial, and a total of 30 trials per group were illustrated. The color scale on the right indicates ΔF/F. D–F) The mean signal before (2 s) and after entry into the drug‐paired chamber (6 s) during pre‐test, post‐test 1 and post‐test 2 (n = 3). The data were presented as the mean ± SEM; One‐way ANOVA and Bonferroni's multiple comparisons test; **p < 0.01, ***p < 0.001, ****p < 0.0001. ΔF/F = (Signal560 nm – Signal410 nm)/ Signal410nm.

In order to investigate whether the neuronal activity in the mPFC was affected by KDM7A and FSCN1, we injected rAAV‐hSyn‐NES‐jRGECO1a‐WPRE‐hGH pA and pAAV‐U6‐shRNA (Kdm7a)‐CMV‐EGFP‐WPRE/ pAAV‐U6‐shRNA (Fscn1)‐CMV‐EGFP‐WPRE or pAAV‐U6‐shRNA (NC)‐CMV‐EGFP ‐WPRE into the mPFC, and implanted optical fibers (**Figure** **7**A). The Ca^2+^ signals of mPFC neurons were recorded with optical fibers during the pre‐test and two post‐tests (Figure 7B). During the pre‐test, there was no significant difference of Ca^2+^ signals among the four groups (Figure 7C,D), indicating that neuronal activity was not influenced by Kdm7a‐shRNA or Fscn1‐shRNA. During post‐test 1, there was a significant increase of neuronal activity in the morphine group after entry into the drug‐paired chamber, compared to the saline group (Figure 7C,E). During post‐test 2, the Ctrl + Morphine group continued to show elevated neuronal activity, while neuronal activity in the Kdm7a‐shRNA + Morphine group and Fscn1‐shRNA + Morphine group showed a significant reduction, compared to Ctrl + Morphine group. These results suggested that knockdown of Kdm7a or Fscn1 blocked morphine‐induced elevation of neuronal activity (Figure 7C,F).

### 2.7 KDM7A Regulates Fscn1 Expression via H3K9me2 and H3K27me2

**Figure 8:**
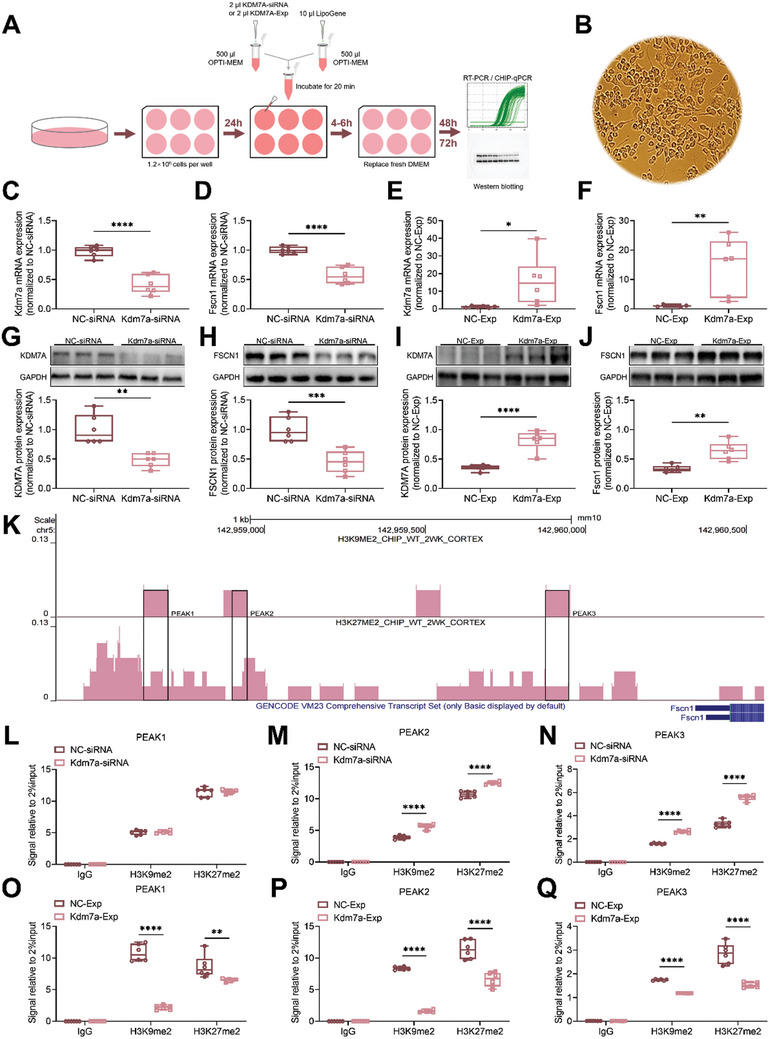
KDM7A regulates Fscn1 expression via H3K9me2 and H3K27me2. A) Transfection of N2a cells with Kdm7a‐specific siRNA/ Exp, the cells were seeded at 50% confluency and transfected with siRNA / Exp duplexes at a final concentration of 50 nM. ChIP‐qPCR and Western blotting were performed after 48 h of transfection. B) N2a cells were visualized under an inverted microscope. C, E, G, I) The transfection of Kdm7a siRNA/ Exp resulted in significant downregulation/ upregulation of Kdm7a mRNA and protein expression in N2a cells (n = 6). The data were presented as the mean ± SEM; t test; *p < 0.05, **P < 0.01, ****p < 0.0001. D, F, H, J) Kdm7a siRNA/ Exp treatment led to reduction/ growth in Fscn1 mRNA and protein expression in N2a cells (n = 6). The data were presented as the mean ± SEM; t test; **p < 0.01, ***p < 0.001, ****p < 0.0001. K) The potential binding sites of H3K9me2 and H3K27me2 at Fscn1 promoter region from the Cistrome Data Browser database, the enrichment index is 0.13. L–N) The ChIP‐qPCR analysis was performed to assess the impact of Kdm7a siRNA and NC siRNA at 3 peak regions of Fscn1 promoter (n = 6). The data were presented as the mean ± SEM; Two‐way ANOVA and Bonferroni's multiple comparisons test. ****p < 0.0001. O–Q) The ChIP‐qPCR analysis was performed to assess the impact of Kdm7a‐Exp and NC‐Exp at 3 peak regions of Fscn1 promoter (n = 6). The data were presented as the mean ± SEM. Two‐way ANOVA and Bonferroni's multiple comparisons test. **p < 0.01. ****p < 0.0001.

Since KDM7A is known as a histone demethylase specific for H3K9me2 and H3K27me2, ChIP was performed to decipher the binding of histone modifications. Kdm7a siRNAs were used to knockdown Kdm7a expression in N2a cells, Kdm7a‐Exp was used to overexpress Kdm7a expression in N2a cells (**Figure** **8**A,B). A non‐targeting siRNA or Exp was used as a control (NC‐siRNA or NC‐Exp). The changes of Kdm7a expression at both mRNA and protein levels was shown in Figure 8C–J. Subsequently, we utilized the Cistrome Data Browser database^[^ ^32^ ^]^ to decipher the potential binding sites of H3K9me2 and H3K27me2 to Fscn1. Through the database,^[^ ^33^ ^]^ we identified three peaks in the promoter region of Fscn1 that could be enriched for H3K9me2 and H3K27me2 (Figure 8K). We designed specific primers for these three peaks and conducted ChIP‐qPCR experiments (**Table** **2**). We first employed ChIP‐qPCR on N2a cells to determine whether KDM7A binds to the Fscn1 promoter region. Our results showed that knockdown of Kdm7a significantly eliminated the binding of KDM7A to Fscn1 promoter (Figure S7, Supporting Information), suggesting KDM7A plays an enzymatic role in regulating Fscn1.

Then we determined the enrichment of H3K9me2 and H3K27me2 at the Fscn1 promoter region in different groups. The results revealed that in PEAK2 and PEAK3, H3K9me2 and H3K27me2 enrichment were significantly upregulated in the Kdm7a siRNA group compared to the NC siRNA group (Figure 8L–N). On the contrary, H3K9me2 and H3K27me2 enrichment were significantly down‐regulated in the Kdm7a‐Exp group compared to the NC‐Exp group (Figure 8O–Q).

To further elucidate how KDM7A regulates Fscn1 through H3K9me2 and H3K27me2 in the morphine addiction model, we conducted additional ChIP‐qPCR experiments on mouse brain tissue. The mice were divided into two groups, Ctrl + Morphine group and Kdm7a‐shRNA + Morphine group. 3 weeks after stereotactic microinjection, morphine‐induced CPP was conducted. On the seventh day of withdrawal, the mPFC tissue was collected, and pooled for ChIP‐qPCR experiments. The results show that the Kdm7a‐shRNA + Morphine group displayed significant upregulation of H3K9me2 and H3K27me2 enrichment compared to Ctrl + Morphine group (Figure S8, Supporting Information).

**Figure 9:**
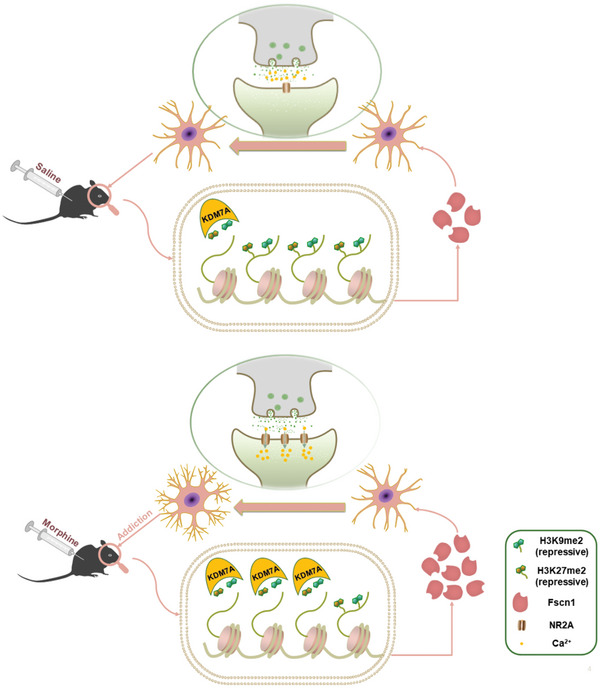
Mechanistic hypothesis diagram. KDM7A targets Fscn1 to regulate synaptic plasticity by removing H3K9me2 and H3K27me2, which serves as a key regulator of morphine‐induced reward memory.

The aforementioned findings provide evidence that the increased binding of the two repressive histone markers, H3K9me2 and H3K27me2 at the promoter region of Fscn1 after Kdm7a knockdown, resulted in the downregulation of Fscn1, which serves as a key downstream gene. Thus, this regulatory mechanism contributes to the modulation of morphine‐dependent memory (**Figure** **9**).

## 3 Discussion

Mounting evidence has illustrated that the KDM7A is associated with different kinds of cancer, inflammation, osteoporosis, and emerging evidence has pointed KDM7A as a critical factor in nervous system, such as normal embryonic and neural development, and neuron differentiation.^[^ ^31^ ^]^ Using a combination of flow cytometry and cell fixation methods, Smith conducted a study in healthy adult rats to sort cells in the central nervous system (CNS).^[^ ^34^ ^]^ Their results indicated that Kdm7a expression was lowest in astrocytes, moderate in microglia, and highest in neurons, suggesting its potential role in neuropsychiatric disorders. Further single‐nucleus transcriptional data from the prefrontal cortex of individuals with varying degrees of Alzheimer's disease revealed differential expression of KDM7A across different pathological stages in six major brain cell types, including excitatory neurons, inhibitory neurons, astrocytes, oligodendrocytes, microglia, and oligodendrocyte progenitor cells.^[^ ^35^ ^]^ Kdm7a knockdown in hippocampus leads to impairment of emotion and memory via repressing neuron activity.^[^ ^31^ ^]^ However, the functional characterization of KDM7A in the adult brain was still largely unknown. In our study, for the first time, we shed light on the involvement of KDM7A in drug‐related reward memory.

### 3.1 KDM7A is a Novel Regulator in Morphine‐Related Reward Memory

KDM7A plays an important role in multiple tissues and organs in the human body (Figure S9A, Supporting Information), with high expression in the cerebral cortex of the brain (Figure S9B–D, Supporting Information). Similar to other KDMs containing PHD and JmjC domains, KDM7A is presumed to regulate neural differentiation and development in mammals. Studies have shown that knockdown of KDM7A impairs neural differentiation, while its overexpression accelerates neural differentiation.^[^ ^36^ ^]^ Inhibition of KDM7 orthologs in zebrafish results in developmental brain defects.^[^ ^15^ ^]^ Furthermore, Kdm7a dysfunction has been implicated in various cancers, including breast cancer,^[^ ^37^, ^38^ ^]^ gastric cancer,^[^ ^39^ ^]^ and prostate cancer.^[^ ^40^ ^]^ Additional investigations have revealed that increased Kdm7a expression suppresses tumor growth in human cancer cells by downregulating angiogenesis through the vascular endothelial growth factor (VEGF) pathway under nutrient starvation.^[^ ^18^ ^]^ However, the underlying mechanisms of KDM7A in the brain remains largely unexplored. Our transcriptome sequencing results demonstrated that Kdm7a knockdown significantly affected the VEGF signaling pathway and cancer‐related pathways, consistent with previous studies. Through overlapping analysis, we revealed the potential involvement of KDM7A in the mPFC in morphine addiction. This analysis showed significant alterations in signaling pathways such as regulation of actin cytoskeleton, cAMP signaling pathway, MAPK signaling pathway, glutamate synapse, and axon guidance following morphine exposure and Kdm7a knockdown. These pathways are critical for drug addiction.^[^ ^41^, ^42^ ^]^ Additionally, our findings highlighted the enrichment of developmental processes in morphine‐associated memory, supporting the notion that addictive drugs “rejuvenate” developmental mechanisms within the brain's reward circuitry, leading to enhanced plasticity in adult differentiated neurons and the formation of drug‐related memories.^[^ ^42^, ^43^ ^]^

CPu is the dorsal part of the striatum, which is a critical structure in the brain's reward circuitry. Neurotransmitters, such as dopamine, modulate the activity of specific brain regions in this circuity and synchronizes the activity to establish the neurobiological mechanism to set the hedonic element of learning.^[^ ^44^ ^]^ Prolonged morphine use results in neuroadaptive changes in the CPu and other brain regions. In the current study, KDM7A expression was downregulated in the CPu after morphine withdrawal. Considering that CPu is significantly innervated by glutamatergic projections from the mPFC and increasing research indicates that this area is involved in drug‐related memories,^[^ ^45^, ^46^, ^47^ ^]^ we speculated that KDM7A‐mediated synaptic plasticity in the CPu relays or coordinates with mPFC to participate in the morphine‐induced memories. However, the specific mechanisms require further exploration.

Previous studies have found that H3K9me2 and H3K27me2 are associated with drug addiction. Chronic intermittent ethanol exposure induced a significant reduction of H3K9me2 in the adult mouse NAc, indicating that this modification might be an effective treatment target to limit relapse vulnerability in drug abusers.^[^ ^48^ ^]^ Besides, both chronic morphine and cocaine could decrease global levels of H3K9me2 in mouse NAc.^[^ ^49^, ^50^ ^]^ Shivakumar also found that exposure to binge‐like ethanol during postnatal day 7 induced neuronal cell loss in rodents, accompanied with increased dimethylation of H3K9me2 and H3K27me2, indicating that dimethylation of histone H3K9 and H3K27 correlates ethanol‐induced neurodegeneration in the developing brain.^[^ ^51^ ^]^

It should be noted that morphine induced a global increase of H3K9me2 in the mPFC on seventh day of morphine withdrawal, while H3K27me2 remain unchanged (Figure S10, Supporting Information). Such inconsistent results revealed complex underlying mechanisms. It is not difficult to speculate that the methylation status of histones is highly dynamic, for example, H3K9me2, shifts from certain genes toward others, therefore, the hypomethylation and hypermethylation could be counteracted on a global basis, emphasizing the importance of gene‐specific modification detection. The current study underscored that demethylation of H3K9me2 and H3K27me2 at the Fscn1 promoter is a critical link in determining morphine‐related memory, supporting the important role of H3K9me2 and H3K27me2 demethylation in drug addiction.

### 3.2 KDM7A Contributes to Morphine‐Induced Synaptic Plasticity by Removing Histone Methylation on Fscn1

The present study made the pioneering discovery of the correlation between KDM7A and FSCN1. FSCN1 is widely expressed during the development of the nervous system (Figure S11A, Supporting Information), both in the human brain and mouse brain (Figure S11B,C, Supporting Information), especially in brain regions such as the cortex (Figure S11D, Supporting Information). According to previous study, Fscn1 exhibited robust expression changes throughout the entirety of the developing CNS in mammals.^[^ ^52^ ^]^ Besides, recent research has shown Fscn1 was upregulated during neurogenesis, indicating potential role of Fscn1 in the formation of mature neurons.^[^ ^53^ ^]^ Robert further proposed that human Fscn1 should be considered as a candidate gene for developmental brain disorders, including mental retardation and autism, especially those for which no obvious neuroanatomical defects are detectable.^[^ ^54^ ^]^ In a phencyclidine‐induced model of schizophrenia, proteomic analysis of mPFC tissue revealed significant elevation of FSCN1 expression.^[^ ^55^ ^]^ Similarly, FSCN1 also exhibited upregulation in Down syndrome.^[^ ^56^ ^]^ The current study illustrated that Fscn1 is essential for morphine‐related memory consolidation and Fscn1 is a downstream target of KDM7A. The expression of Fscn1 is regulated by the demethylation of two inhibitory histone modifications, H3K9me2 and H3K27me2.

As a special kind of cell protrusions, dendritic spines are small protrusions (<2 µm) arising from dendrites of most neurons in the CNS. Dendritic shafts are covered by thin, long protrusions called dendritic filopodia, which are thought to be precursors of spines. Dendritic spines show actin‐based rapid motility in the time scale of seconds. This motility could be represented by extensive length changes, including both extension and retraction.^[^ ^57^ ^]^ Dendritic spines were demonstrated to be with high plasticity, capable of both formation and elimination.^[^ ^58^ ^]^ Their structural dynamics are intimately linked to dendritic strengthening or weakening.^[^ ^59^, ^60^, ^61^ ^]^ Hence, alterations in the number and configuration of dendritic spines serve as indicators of neural activity.^[^ ^62^ ^]^ Exposure to amphetamine, cocaine, nicotine, or morphine produces persistent changes in the structure of dendrites and dendritic spines in brain regions involved in motivation, reward, judgment, and inhibitory control of behavior.^[^ ^63^ ^]^ Specifically, repeated exposure to morphine causes long‐term changes in the density of postsynaptic sites (dendrites and spines) in sensitive brain regions such as the prefrontal cortex, limbic system (hippocampus, amygdala), caudate and NAc.^[^ ^64^ ^]^ Ayantika Pal discovered that after chronic morphine treatment and withdrawal, there was a significant increase of dendritic spine density in the frontal cortex compared to the untreated control group. This increase was accompanied by an elevation of Shank1 protein levels in both the cortex and midbrain. It is worth mentioning that overexpression of Shank1 has been found to promote dendritic spine maturation and growth,^[^ ^65^ ^]^ which is consistent with the role of Fscn1 in synapse pruning.^[^ ^66^ ^]^ During chronic cocaine addiction, the dendritic spine density of medium spiny neurons significantly increased at both the shell and core regions of the NAc.^[^ ^67^, ^68^ ^]^

The growth of axons and dendrites is a dynamic process which is crucial for neuronal development. In this process, growth cones, which are specialized structures and located at the tips of extending neurites, play a pivotal role in sensing and responding to signals from the surrounding environment to guide neuronal extension and connectivity. FSCN1 is abundantly expressed in growth cones to control stability. When FSCN1 is inactivated, the stability of actin filaments is compromised, leading to the instability and collapse of growth cones.^[^ ^69^ ^]^ Besides, the rapid clearance of dying neurons and non‐functional synapses in the brain is performed by microglia, and FSCN1 is involved in striatal microglia clearance. Aberrant clearance has a negative impact on neuronal morphology and function, such as decreased learning and memory, which are often associated with neurodegenerative and psychiatric disorders.^[^ ^70^ ^]^ In summary, FSCN1 plays a crucial role in neuronal structure and morphology, however, the driving force to initiate this process remain largely unknown. The current study demonstrated morphine exposure led to the upregulation of the KDM7A in the mPFC, which triggers the increased expression of downstream Fscn1 gene. The NR2A is a critical subunit of the NMDA receptor in synapses. NMDA receptors, including those containing the NR2A subunit, are primarily located on the postsynaptic membrane of neurons. Changes in the expression or activity of the NR2A subunit can alter the strength of synaptic transmission. For instance, the absence or dysfunction of NR2A may lead to abnormalities in synaptic plasticity, thereby impacting learning and memory functions.^[^ ^71^, ^72^, ^73^ ^]^ Results in the current study showed that NR2A expression was enhanced after morphine withdrawal, and knockdown of Fscn1 reversed this morphine‐induced increase of NR2A. Therefore, knockdown of Fscn1 disrupted morphine‐induced memory and synaptic plasticity at least partly through inhibiting NR2A in the mPFC, thereby impeding neurotransmission and ultimately causing a decrease in neuronal activity. These findings strongly support the critical roles of KDM7A and FSCN1 in controlling dendritic spine plasticity at the context of morphine addiction. Additionally, KDM7A and FSCN1 could serve as potential therapeutic targets, providing clues for the development of new intervention strategies and drug treatments. Further research can explore the detailed functions and interrelationships of these genes, as well as their specific roles in drug‐related behavior and dendritic spine plasticity.

In summary, our findings demonstrated that KDM7A in the mPFC may exert regulatory control over the transcription of Fscn1 by modulating the demethylation processes associated with H3K9me2 and H3K27me2 in the Fscn1 promoter region. This regulatory mechanism specifically influenced the density of neuronal dendritic spines. Significantly, KDM7A in the mPFC plays a crucial role in morphine‐dependent memory in mice.

## 4 Experimental Section

### Animals and Drugs

Male C57BL/6 mice aged 8–10 weeks were utilized for the experiments, and they were obtained from the Experimental Animal Center of Xi'an Jiaotong University. The mice were group‐housed, with four animals per cage, and provided ad libitum access to food and water. A 12‐h light/12‐h dark cycle was maintained in the animal facility. All behavioral tests were conducted during the light cycle. The experimental procedures strictly adhered to the guidelines outlined in the National Institutes of Health Guide for the Care and Use of Laboratory Animals and were approved by the Animal Care and Use Committee of Xi'an Jiaotong University (No. 2019–901). Morphine, obtained from the National Institutes for Food and Drug Control (China), was dissolved in 0.9% saline and administered intraperitoneally (i.p.) at a dosage of 10 mg kg^−1^ for conditioning and 5 mg kg^−1^ for reinstatement.

### Conditioned Place Preference

The CPP test was conducted following a previously described protocol with some modifications.^[^ ^74^, ^75^, ^76^, ^77^ ^]^ The CPP apparatus consisted of a 3‐compartment box, with two conditioning compartments that were identical in size. However, one chamber was distinguished by black walls and a rectangular net on the floor, while the other chamber had white walls and perforated holes on the floor. These visual and tactile cues provided distinct environmental contexts. The two conditioning compartments were separated from the neutral chamber by removable boards.

Before the CPP session, behavioral subjects were individually habituated to the investigator through 10 min of daily handling for 1 week. In the pre‐test phase, mice were given access to both chambers for 15 min, and their time spent in each chamber was recorded. During the conditioning phase, mice received either morphine (10 mg kg^−1^, i.p.) or saline and were confined to one chamber for 45 min. On the following day, mice were injected with either saline or morphine, which they had not previously received, and immediately placed in the other chamber for another 45 min. Specifically, morphine was injected on Day 2, 4, 6, 8, 10. The 10‐day duration (5 cycles) allows ample time for animals to develop very stable memory. This duration allows animals with sufficient exposure to drug‐related contexts. Meanwhile, it minimizes potential confounding factors or habituation effects that could arise from excessively long testing periods. For reinstatement, all the mice received post‐test 3 immediately after morphine (5 mg kg^−1^, i.p.) injection.

### Quantitative RT‐PCR

RNA was extracted from isolated tissues of bilateral mPFC, NAc, CPu, and hippocampus using the Simply P Total RNA Extraction Kit (BSC60S1, BioFast) following the manufacturer's recommended protocol. The quantity of RNA was determined spectrophotometrically using the NanoDrop 2000c, and then reverse transcribed into complementary DNA (cDNA) using the PrimeScript RT Master Mix (TaKaRa) for subsequent qRT‐PCR analysis (Invitrogen).

Real‐time quantitative PCR was performed using the TB Green Premix Ex Taq II (Tli RNaseH Plus) (Code No. RR820A, TaKaRa). The primer sequences used in the experiments are detailed in **Table** **1**. The analysis of the qRT‐PCR data was carried out using the 2^−(∆∆Ct)^ method, which allows for the comparison of relative gene expression levels.

**Table 1:** Primer sequences for qRT‐PCR.

Genes	Sequence
*Kdm7a*	F:GCTTGGTTTCATGCCAGATAAG
	R:TGATAGGTGGTGGGTTTGATG
*Gapdh*	F:TCTCCTGCGACTTCAACA
	R:TGTAGCCGTATTCATTGTCA
*Fscn1*	F:AGGATGAAGAGACCGATCAGG
	R:CCACTCGATGTCAAAGTAGCAG

### Western Blotting Analysis and Antibodies

To quantify proteins, tissues containing the mPFC, NAc, CPu, and hippocampus were isolated from animals and immediately flash‐frozen on dry ice. The frozen tissue was then lysed in SDS lysis buffer, followed by centrifugation at 12 000 rpm at 4 °C for 15 min to collect the supernatant. Protein concentration in the samples was determined using the Omni‐Easy Instant BCA Protein Assay Kit (Epizyme, ZJ102). Samples were diluted in sample buffer, and was loaded on 10% polyacrylamide gels supplemented with β‐mercaptoethanol and bromophenol blue. The gels were transferred onto polyvinylidene difluoride (PVDF) membranes (Millipore) for 2 h on ice. The membranes were then blocked in 5% milk in TBST (20 mM Tris pH 7.5, 150 mM NaCl, 0.1% Tween‐20) for 1 h at room temperature. Subsequently, the membranes were incubated overnight at 4 °C with the primary antibodies. After washing the membranes in 5% TBST, they were incubated with the secondary antibody (1:10 000) at room temperature. Goat anti‐Rabbit IgG (PIONEER Biotechnology, 31 460) secondary antibodies was used. Following the antibody incubation, the protein bands were visualized using enhanced chemiluminescence (ECL) with the SuperSignal West Pico chemiluminescent substrate (Thermo Fisher Scientific). The primary antibodies were shown as follows: KDM7A (Genetex, GTX32688, 1:500), FSCN1 (Proteintech, 14384‐1‐AP, 1:5000), and GAPDH (Abcam, ab263962, 1:1000).

### Stereotaxic Surgeries

The mice were anesthetized with 0.3% sodium pentobarbital (30 mg kg^−1^) for surgical procedures. The mouse brain was secured using a stereotaxic apparatus (RWD life science) to ensure precise targeting. Viral injections were specifically aimed at mPFC region, following coordinates (AP, +2.0 mm; ML, ± 0.3 mm; DV, −2.3 mm) provided by the Paxinos and Franklin mouse brain atlas (Paxinos G, 2008). For intracerebral infusion, a volume of 400 nL of the viral vector was bilaterally injected into the mPFC. The solution was injected at a rate of 50 nL min^−1^, and the injection cannula was left in place for an additional 10 min to minimize backflow.

For behavioral tests, following the injections, mice were given a recovery period of 21 days before behavioral test. At the end of the behavior experiments, the injection sites were confirmed. Animals with inaccurate or off‐target injection sites were excluded from data analysis to ensure the validity of the results.

### Morris Water Maze

The MWM was conducted 3 weeks after Kdm7a‐shRNA or NC‐shRNA microinjection into the mPFC. The protocol was performed as previously described with some modifications.^[^ ^78^ ^]^ A hidden platform was placed 1 cm under water in the target quadrant. An overhead camera was positioned above the tank to automatically monitor each animal's performance using a video‐computerized tracking system (SMART, Panlab SL, Barcelona, Spain). During the learning phase, mice were randomly placed in the water at one of four fixed entry points facing the tank wall. If a mouse reached the platform within 60 seconds, it would be allowed to stay on the platform for 10 seconds. The mice would be guided to stay on the platform for 15 s if they did not find the platform in 1 min, with the latency recorded as 60 seconds. The average latency and speed across the four daily trials were used as key indicators of learning capability. Mice were trained four times a day for 5 consecutive days. The probe tests were performed 24 h and 7 days after the final training trial. During the probe test phase, the mice were placed in the water farthest from the platform and recorded for 60 seconds without the platform. Then the number of platform crossings, time spent in the target quadrant, and time spent in the original platform location were recorded.

### RNA Sequencing and Data Analysis

RNA was extracted from the mouse mPFC tissue. For cDNA library preparation, 1 µg of total RNA was used following the protocol of cDNA‐PCR Sequencing Kit (SQK‐PCS109) provided by Oxford Nanopore Technologies (ONT). The full‐length cDNAs were enriched by the addition of defined PCR adapters to both ends of the first‐strand cDNA. Briefly, cDNA amplification was performed using the LongAmp Taq (NEB). The PCR products were subsequently ligated with ONT adaptor ligation using T4 DNA ligase (NEB). The DNA was purified using Agencourt AMPure XP beads (Beckman) in accordance with the ONT technique. The final cDNA libraries were loaded onto FLO‐MIN109 flow cells and sequenced on the PromethION platform. The processing of data analysis began with quality control. Raw reads were filtered with a minimum quality score threshold of 7 and a minimum length criterion of 500 base pairs (bp). Subsequently, ribosomal RNA (rRNA) sequences were eliminated after being aligned to rRNA database. Then, full‐length, non‐chimeric transcripts were identified by searching for primer sequences at both ends of the filtered reads. These FLNC transcripts were clustered by mapping to the reference genome (mm 10) using mimimap2, and the consistency order was obtained using Pinfish. The resulting consensus sequences were then mapped to the reference genome for further analysis. Utilizing the cDNA Cupcake package, the mapped reads were consolidated further, applying a minimum coverage threshold of 0.85 and a minimum identity criterion of 0.90. Reads with a match quality exceeding 5 were selectively employed for quantification. Expression levels were conducted by assessing the number of reads per gene/transcript relative to 10 000 mapped reads. For differential expression analysis, the edgeR software was harnessed to identify transcripts with significant changes in expression, defined as having an absolute fold change of at least 1.50 and FDR below 0.05. Enrichment analysis was conducted on the differential transcripts, including functional annotation by GO database.

### Golgi‐Cox Staining

Golgi‐Cox staining was employed to visualize dendritic spines of mPFC sections. Initially, the tissue was placed in a mixture of Solution A (containing potassium dichromate and mercuric chloride) and Solution B (containing potassium chromate). After 24 h, the chromate solution was replaced, and the tissue slices were kept in the dark in the chromate solution for 5 days.

Subsequently, the tissue was immersed in a mixture of Solution C (containing NaH_2_PO_4_, Na_2_HPO_4_, and NaCl) and Solution D (containing sucrose, PVP40, and ethylene glycol) for 24 h. This solution was then replaced and maintained at 4 °C for another 5 days. The tissue slices, with a thickness of 100 µm, were mounted on gelatin‐coated slides (75 × 25 mm). The slides were left to dry in the dark for 2 h.

To prepare the slides for visualization, they were soaked, along with the slide holder, in distilled water twice for 5 min each time. Subsequently, the slides were soaked in 50% ethanol for 5 min, followed by an 8‐min soak in a solution of 30% ammonia. After rinsing, the slides were then soaked in a 5% sodium thiosulfate solution in the dark for 10 min. After rinsing, the slices were dehydrated using graded alcohols (70%, 95%, and 100% ethanol in deionized water) and cleared with xylenes. Finally, the slides were sealed with Permount Toluene Solution.

### Fiber Photometry

Mice were unilaterally injected with 200 nL of rAAV‐hSyn‐NES‐jRGECO1a ‐WPRE‐hGH pA and 200 nL of pAAV‐U6‐shRNA (Kdm7a/ Fscn1)‐CMV‐EGFP‐WPRE or pAAV‐U6‐shRNA (NC)‐ CMV‐EGFP‐WPRE. Mice were implanted with a unilateral optical fiber (400 µm core, 0.39 numerical aperture (NA), RWD, China). 3 weeks after surgery, fiber photometry recordings were performed using a multiplex photometry system (R810, RWD Life Science, China). Three‐phase cycling of 410, 470, and 560 nm LEDs were used to record the fluorescence when the mice shuttle through the CPP box. ΔF/F = (Signal560 nm – Signal410 nm)/ (Signal 410 nm). On the day of the CPP behavioral test, after recording the baseline fluorescence with optical fiber for 3 min, the Ca^2+^ signal changes of mPFC in mice entering the black chamber or white chamber within 15 min during the pre‐test and post‐test were monitored.

### Cell Culture and siRNA/ Exp Transfection

N2a cells were obtained from Procell Life Science & Technology (Wuhan, China). The N2a cells were cultured in Dulbecco's Modified Eagle's Medium supplemented with 10% fetal bovine serum (C11995500BT, Gibco, Thermo Fisher Scientific, California, USA), 100 U mL^−1^ penicillin, and 100 µg mL^−1^ streptomycin. The cells were maintained at 37 °C in a humidified atmosphere containing 5% CO_2_. The cell lines were grown until they reached 80% confluence and were then passaged using trypsin at a ratio of 1:4. SiRNA/ Exp specifically targeting Kdm7a and control siRNA/ Exp were obtained from TSINGKE (Beijing, China). For Kdm7a siRNA/ Exp transfection, the cells were seeded at 50% confluency and transfected with siRNA/ Exp duplexes at a final concentration of 50 nM using Hieff Trans Liposomal Transfection Reagent (Yeasen, Shanghai, China) following the manufacturer's protocol. The transfection was carried out for 48 h.

### Chromatin Immunoprecipitation‐Quantitative Real‐Time PCR (ChIP‐qPCR)

ChIP was conducted in N2a cells to investigate epigenetic modifications. N2a cells (4 × 10^6^ per IP) were plated in 10‐cm culture dishes and cross‐linked with 1% formaldehyde, followed by quenching with glycine. The cells were then lysed, and the nuclei were treated with micrococcal nuclease at 37 °C for 20 min. The reaction was halted using 0.5 mol L^−1^ EDTA, and the samples were sonicated to disrupt the nuclear membrane. After centrifugation, the supernatant containing the chromatin was collected.

The chromatin solution was incubated separately with antibodies against specific histone modifications, including anti‐H3K9me2 (Abcam, ab1220, 2–4 µg for 25 µg of chromatin), anti‐H3K27me2 (CST, 9728S, 1:50), anti‐Histone H3, and anti‐normal rabbit IgG (CST, USA). The mixture was rotated overnight at 4 °C, followed by incubation with ChIP‐grade protein G magnetic beads at 4 °C. The beads were washed to remove non‐specific binding. The cross‐links were reversed by heating at 65 °C for 2 h, and the DNA was purified for subsequent ChIP‐qPCR analysis. For the ChIP‐qPCR experiments, specific pairs of primers for 3 peaks were designed targeting the promoter region of Fscn1. The primer sequences are listed in Table 2.

**Table 2:** Primer sequences for ChIP‐qPCR.

Primer	Sequence
*Primer1*	F:CTCCGGCCTTTACCTCTGGTGT
	R:CCGTGAACGCTCTGTATTGATTAG
*Primer2*	F:TGGGTTAAACAGGTTTGAACAGAG
	R:CTTGAGGGAGCAGGTCAGAAGA
*Primer3*	F:GCTTCTTCAAGGACCGGCTCTA
	R:GTCGATGGGAAGGACCAGGTTT

Real‐time PCR was performed to detect the ChIP‐generated DNA, and the data were normalized to the 2% input control.

### Statistical Analysis

All statistical analyses were performed using SPSS statistics 24. Normality was assessed using the Shapiro‐Wilk test, and homogeneity of variances was tested using Levene's test. The outliers were identified using Box plots, and typically defined above the third quartile or below the first quartile. For normally distributed and homoscedastic data, two‐group comparisons were conducted using Unpaired t test. One‐way ANOVA was used for comparisons among multiple groups with one factor. Two‐way ANOVA was employed for comparisons among multiple groups with two factors, followed by Bonferroni's post‐hoc test. Mann‐Whitney U test was applied for data not conforming to normal distribution. The significance level was set at *p* < 0.05. The sample size (n) generally ranges from 3 to 10, and the specific n value for each analysis was provided in the figure legend. Data were presented as mean ± SEM. All figures were prepared using Adobe Illustrator or GraphPad Prism. Significance is denoted as ^#/^*p < 0.05, ^##/^**p < 0.01, ^###/^***p < 0.001 and ^####/^****p < 0.0001 in the figures.

### Electronic Resources

 

GTEx Portal: https://www.gtexportal.org/home
MGI: http://www.informatics.jax.org/
OMIM: http://www.omim.org
SFARI Gene: https://gene.sfari.org/database/human‐gene/
UCSC Genome Browser: http://genome.ucsc.edu
Cistrome DB: http://cistrome.org/db/#/

## Conflict of Interest

The authors declare no conflict of interest.

## Supporting information



Supporting Information

## Data Availability

The data that support the findings of this study are available from the corresponding author upon reasonable request. Sequence data can be accessed through the Gene Expression Omnibus (GEO) under the NCBI accession number GSE276730.
