# Supplementary material for: The Histone Lysine Demethylase KDM7A Contributes to Reward Memory via Fscn1‐Induced Synaptic Plasticity in the Medial Prefrontal Cortex
Source: Adv Sci (Weinh). 2025 Jan 21;12(10):2405352. doi: 10.1002/advs.202405352 (PMC11905110; doi:10.1002/advs.202405352)
Supplement: Supplementary file 1 — Supporting Information [file ADVS-12-2405352-s001.docx]

Supporting Information

**The Histone Lysine Demethylase KDM7A Contributes to Reward Memory via Fscn1-induced Synaptic Plasticity in the Medial Prefrontal Cortex**

Zhuo-jin Yang^1,3^, Dong-yu Yu^1,3^, Fei-fei Gao^1^, Dan-ya Zhou^2^, Ya-nan Wu^1^, Xi-xi Yang^1^, Jie Chen^1^, Jing-si Yang^1^, Meng-qing Shen^1^, Yu-xiang Zhang^1*^, Lai Wei^2*^, Chun-xia Yan^1*^

**Table S1**

**Figure S1**

**Figure S2**

**Figure S3**

**Figure S4**

**Figure S5**

**Figure S6**

**Figure S7**

**Figure S8**

**Figure S9**

**Figure S10**

**Figure S11**

**Table S1. Quantification and differential expression analysis of transcripts**

| DET set | DET number | up-regulated | down-regulated |
| --- | --- | --- | --- |
| Ctrl + Morphine *vs.* Kdm7a-shRNA + Morphine | 2366 | 1368 | 998 |
| Ctrl + Saline *vs.* Ctrl + Morphine | 387 | 265 | 122 |
| Ctrl + Saline *vs.* Kdm7a-shRNA + Saline | 5754 | 3140 | 2614 |
| Kdm7a-shRNA + Saline *vs.* Kdm7a-shRNA + Morphine | 57 | 19 | 38 |

**Supplementary Figures**


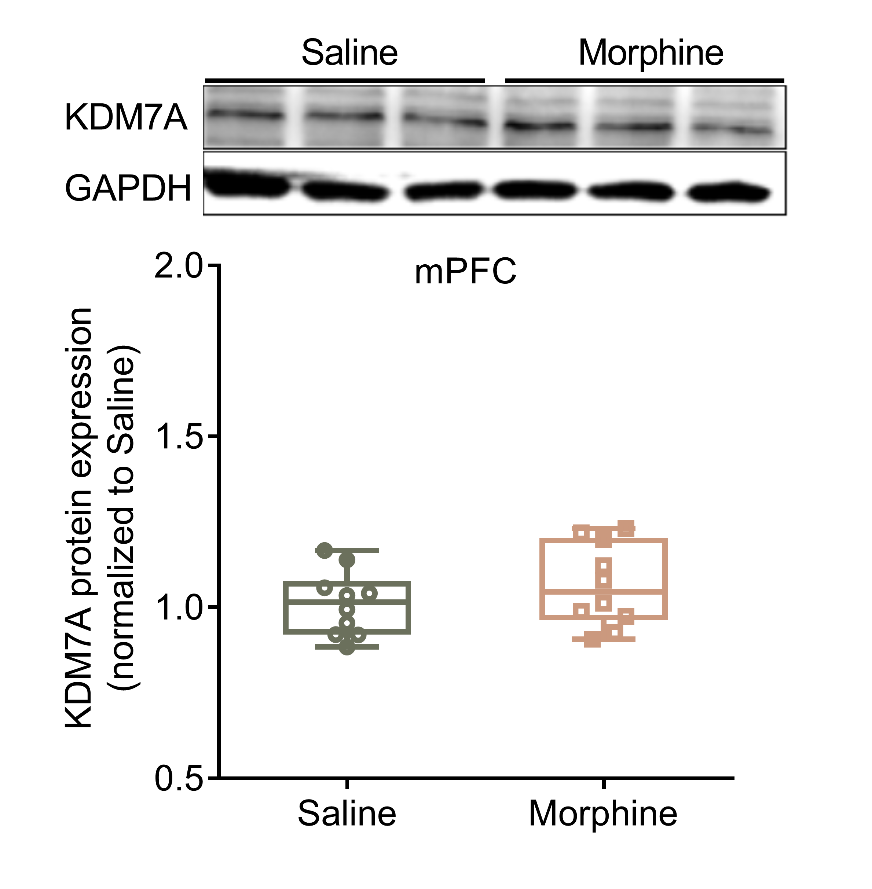


**Figure S1. No significant effect of morphine on KDM7A protein expression in the mPFC on Day 30.** n = 10. The data were presented as the mean ± SEM. Unpaired t-test.


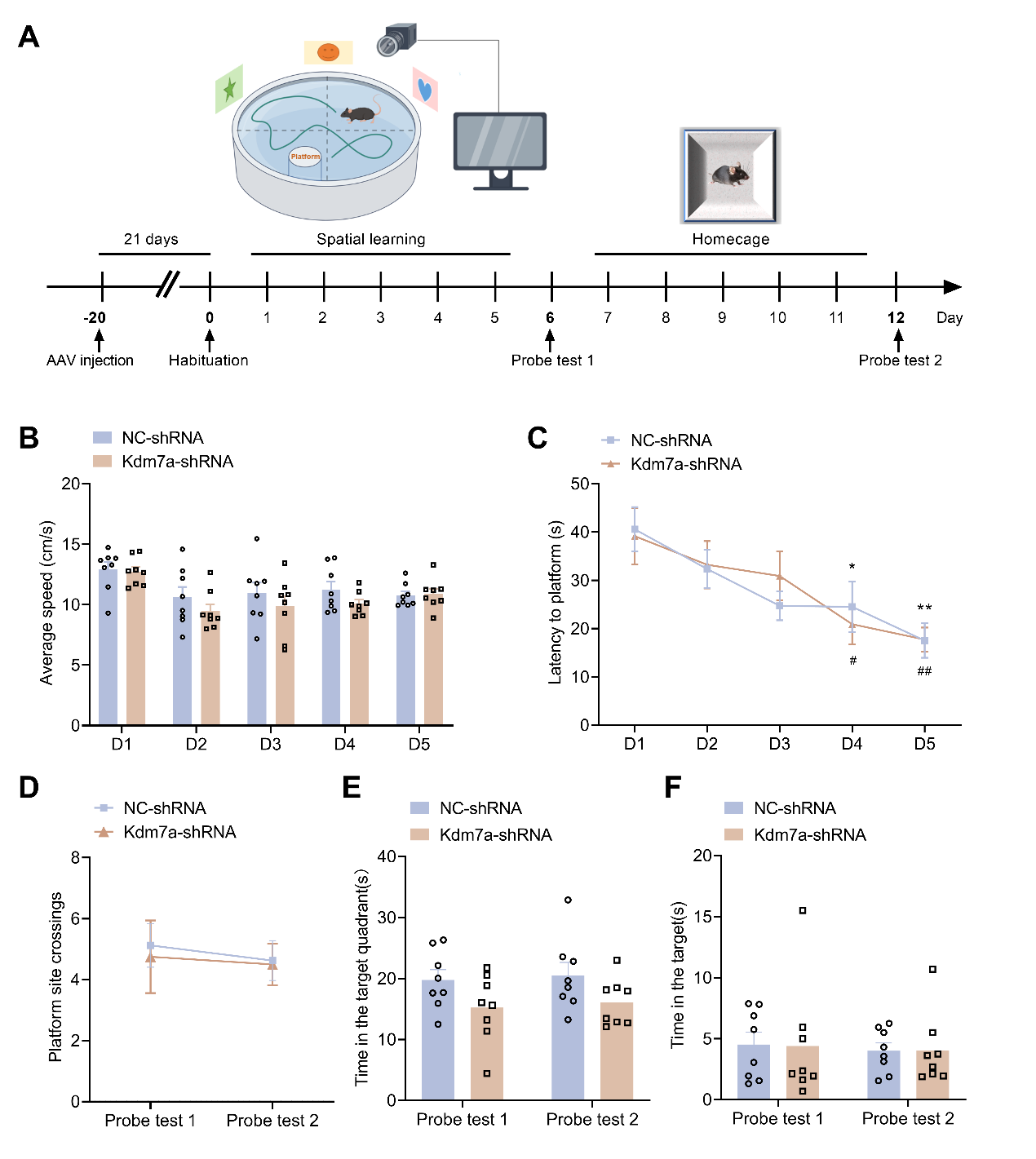


**Figure S2. Kdm7a knockdown had no effect on spatial learning and memory.** A) The timeline of Morris water maze test. B) Mean swimming speed during learning phase (n = 8). The data were presented as the mean ± SEM; Two-way ANOVA and Bonferroni’s multiple comparisons test. C) Latency to the platform during learning phase (n = 8). The data were presented as the mean ± SEM; Two-way ANOVA and Bonferroni’s multiple comparisons test. **P* < 0.05, ***P* < 0.01 (Kdm7a-shRNA group compared with D1). ^#^*P* < 0.05, ^##^*P* < 0.01 (NC-shRNA group compared with D1). D- F) The number of platform site crossings, time in the target quadrant, and time in the original platform area (n = 8). The data were presented as the mean ± SEM; Two-way ANOVA and Bonferroni’s multiple comparisons test.


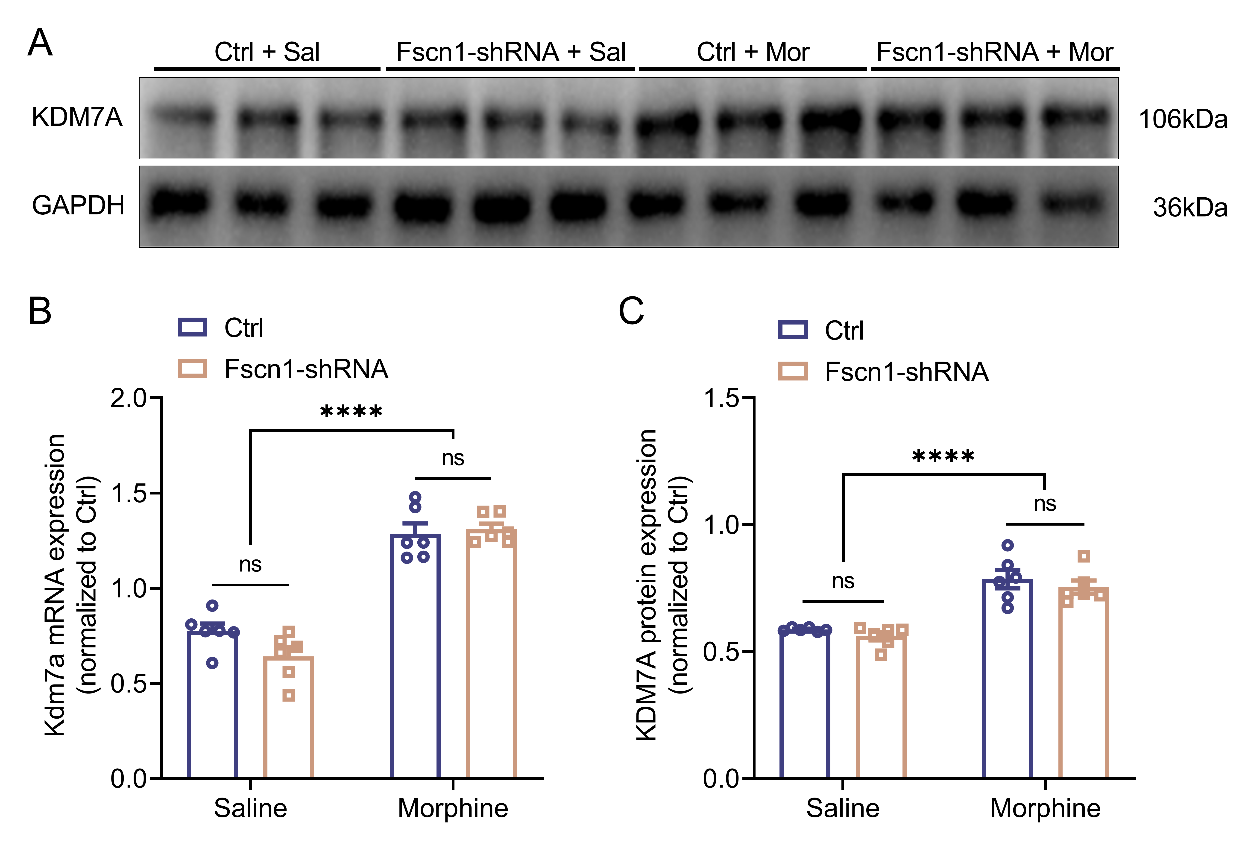


**Figure S3. Fscn1 knockdown had no effect on the expression of KDM7A.** A, C) The expression of KDM7A protein (n = 6). The data were presented as the mean ± SEM; Two-way ANOVA and Bonferroni’s multiple comparisons test. *****P* < 0.0001. B) The expression of Kdm7a mRNA (n = 6). The data were presented as the mean ± SEM; Two-way ANOVA and Bonferroni’s multiple comparisons test. *****P* < 0.0001.


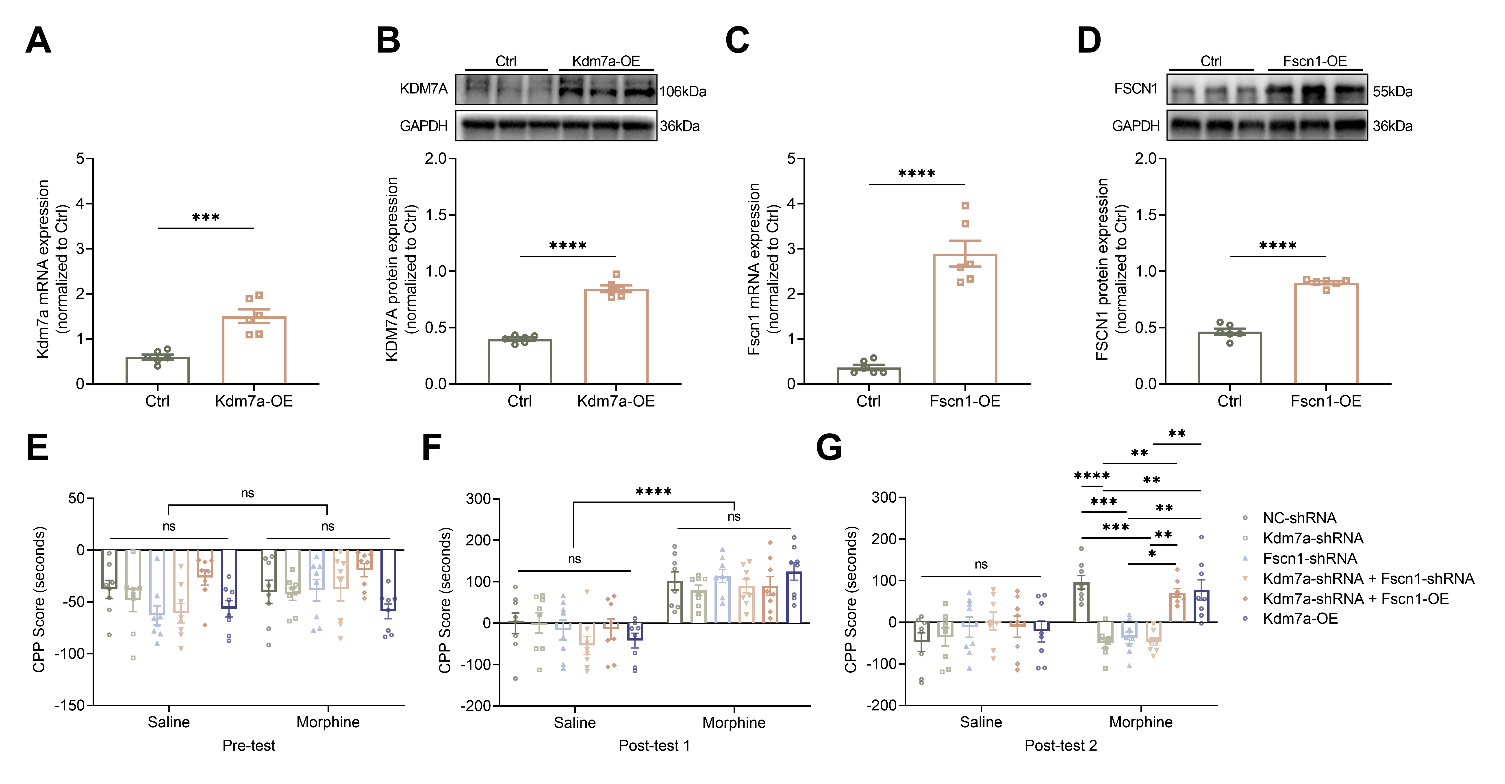


**Figure S4. Double knockdown of Kdm7a and Fscn1 attenuated memory consolidation.** A) Kdm7a mRNA expression in the mPFC upregulated after Kdm7a overexpression (n = 6). The data were presented as the mean ± SEM; Unpaired t test; ****P* < 0.001. B) Significant increase of KDM7A protein expression in Kdm7a overexpression virus group compared to Ctrl virus group (n = 6). The data were presented as the mean ± SEM; Unpaired t test; *****P* < 0.0001. C) Fscn1 mRNA expression in the mPFC decreased after Fscn1 overexpression (n = 6). The data were presented as the mean ± SEM; Unpaired t test; *****P* < 0.0001. D) Significant increase of FSCN1 protein expression in Fscn1 overexpression virus group compared to Ctrl virus group (n = 6). The data were presented as the mean ± SEM; Unpaired t test; *****P* < 0.0001. E-G) CPP score of pre-test, post-test 1 and post-test 2 (n = 8). The data were presented as the mean ± SEM; Two-way ANOVA and Bonferroni’s multiple comparisons test. **P* < 0.05, ***P* < 0.01, ****P* < 0.001, *****P* < 0.0001.

**
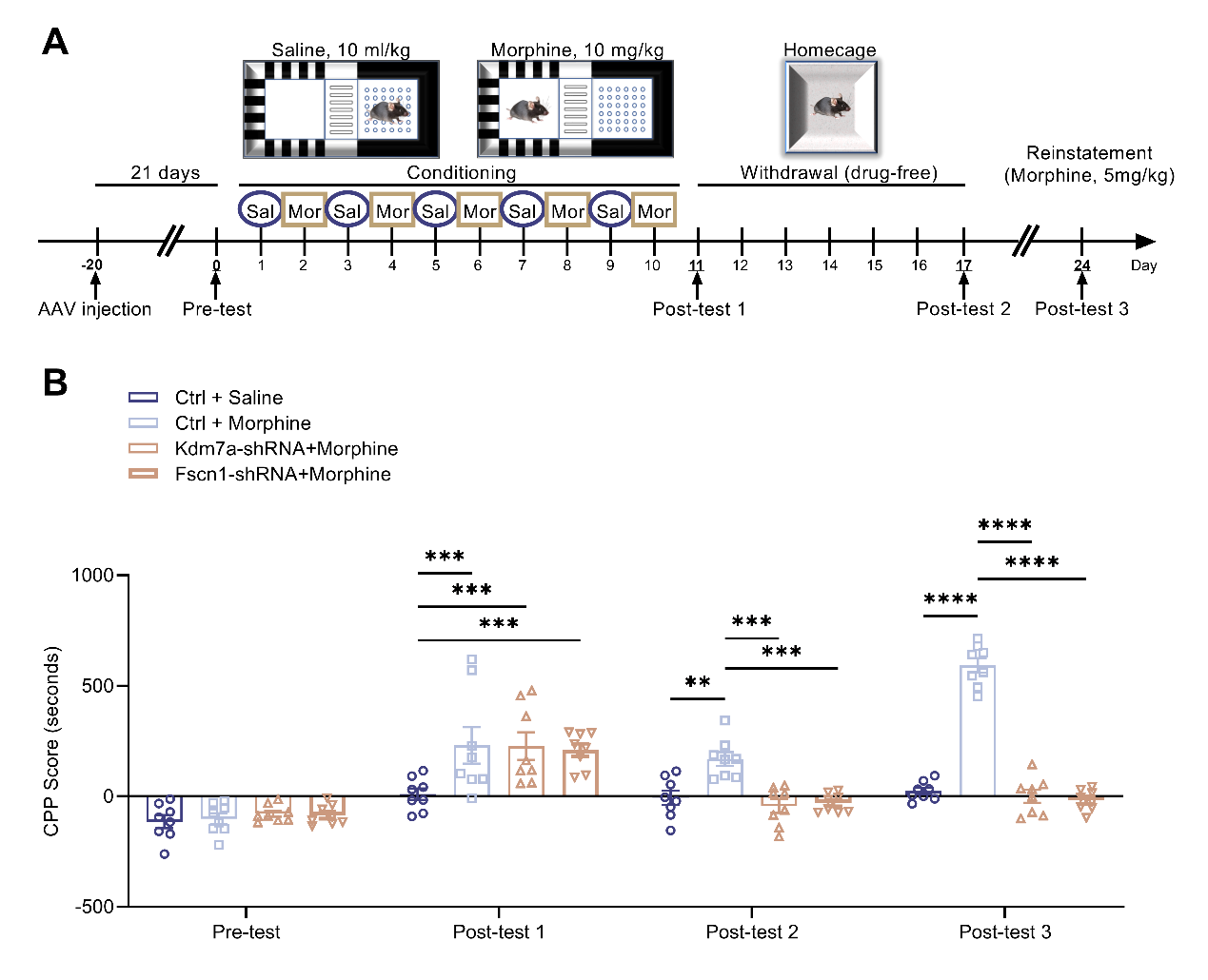
Figure S5. Kdm7a or Fscn1 knockdown attenuated morphine-primed reinstatement.** A) The timeline of CPP paradigm. On Day 24, morphine (5mg/kg) was injected to all the mice. B) CPP score of pre-test, post-test 1, post-test 2 and post-test 3 (n = 8). The data were presented as the mean ± SEM; Two-way ANOVA and Bonferroni’s multiple comparisons test. ***P* < 0.01, ****P* < 0.001, *****P* < 0.0001.


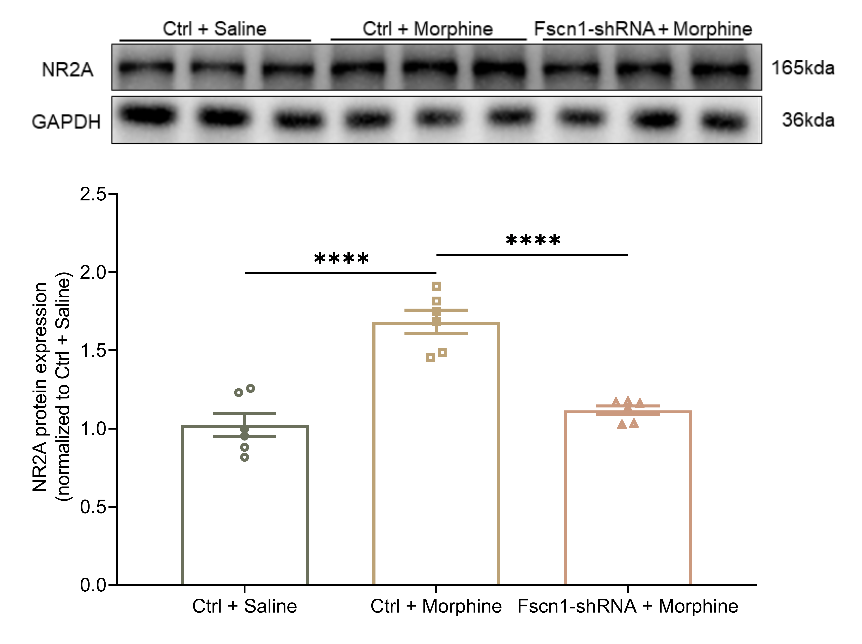


**Figure S6. Knockdown of Fscn1 reversed morphine-induced increase of NR2A.** n = 6. The data were presented as the mean ± SEM; One-way ANOVA and Bonferroni’s multiple comparisons test; *****P* < 0.0001.
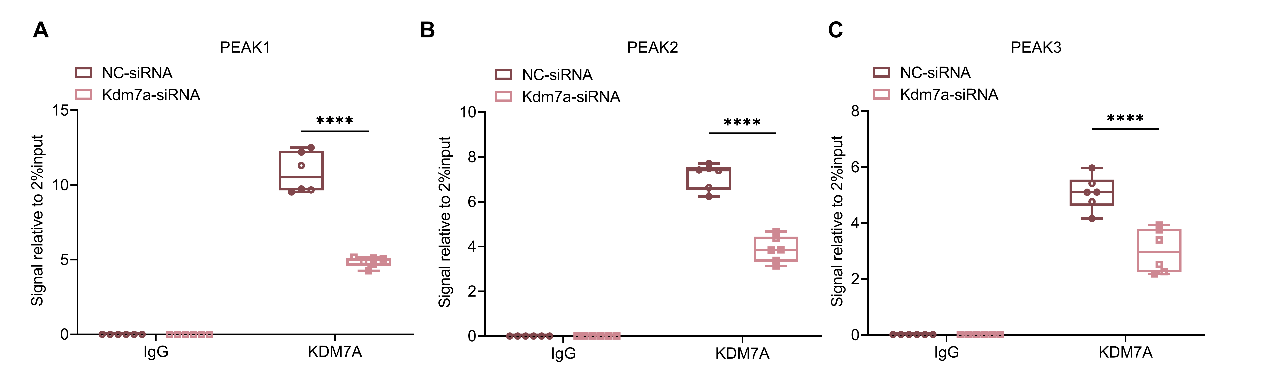


**Figure S7. Knockdown of Kdm7a significantly eliminated the binding of KDM7A to Fscn1 promotor.** A-C) The ChIP-qPCR analysis showing the enrichment of KDM7A at 3 peak regions of Fscn1 promotor (n = 6). The data were presented as the mean ± SEM; Two-way ANOVA and Bonferroni’s multiple comparisons test. *****P* < 0.0001.


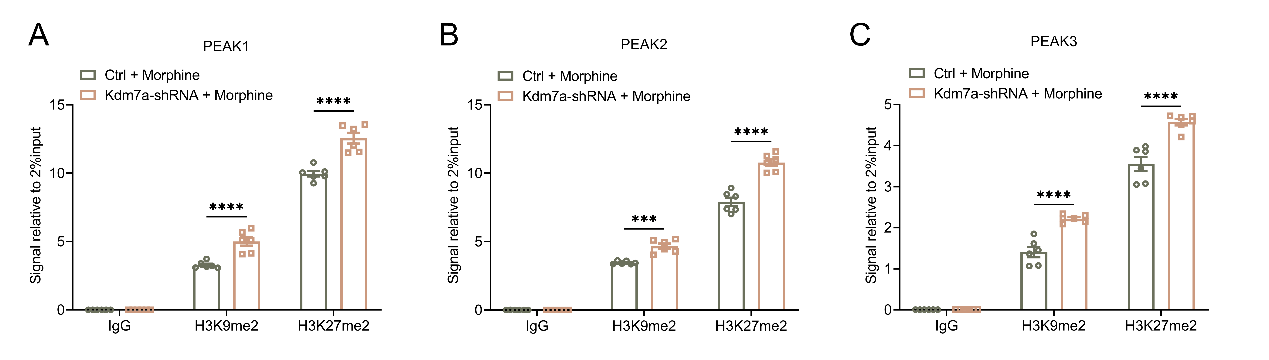


**Figure S8. KDM7A regulates Fscn1 expression via H3K9me2 and H3K27me2.** A-C) The ChIP-qPCR analysis of mouse mPFC after Kdm7a knockdown to assess the enrichment of H3K9me2 and H3K27me2 at 3 peak regions of Fscn1 promotor (n = 6). The data were presented as the mean ± SEM; Two-way ANOVA and Bonferroni’s multiple comparisons test. ****P* < 0.001, *****P* < 0.0001.


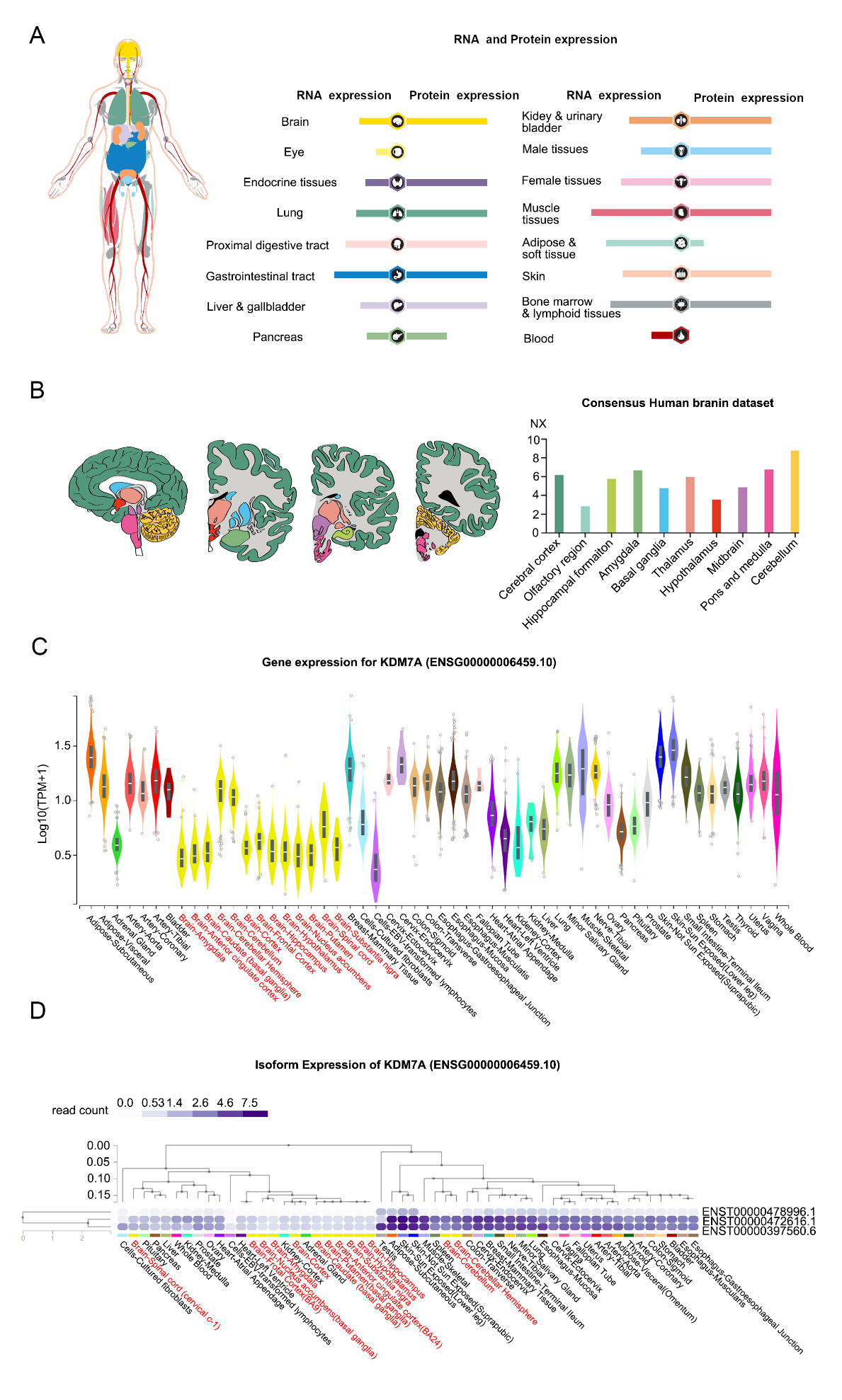


**Figure S9. The mRNA and protein expression of KDM7A in the human central nervous system.** A) RNA and protein expression data generated in the Human Protein Atlas project. Analyzed tissues are divided into color-coded groups according to which functional features they have in common. RNA expression summary shows the consensus RNA-data based on normalized expression (NX) data from three different sources: internally generated Human Protein Atlas (HPA) RNA-seq data, RNA-seq data from the Genotype-Tissue Expression (GTEx) project and CAGE data from FANTOM5 project. Protein expression score is based on a best estimate of the "true" protein expression from a knowledge-based annotation, described in the Human Protein Atlas project. B) Consensus Human brain dataset generated in the Human Protein Atlas project. Consensus normalized expression (NX) levels were created for the 10 brain regions by combining the data from two transcriptomics datasets (GTEx and FANTOM5). Color coding is based on brain region and the bar shows the highest expression among the subregions included. C) Gene expression for KDM7A (ENSG00000006459.10) generated in the GTEx Portal. The data used for the analyses were obtained from the GTEx Portal on 30/4/24 and dbGaP accession number phs000424.v8.p2 on 30/4/24. Expression values are shown in TPM (Transcripts Per Million) calculated from a gene model with isoforms collapsed to a single gene. Box plots are shown as median and 25th and 75th percentiled, and points are displayed as outliers if they are above or below 1.5 times the interquartile range. D) Isoform expression of KDM7A (ENSG00000006459.10) generated in the GTEx Portal. In the isoform expression view, the median expression of each isoform in each tissue is summarized using a heatmap. The tissues (rows) and isoforms (columns) are ordered based on hierarchical clustering using the Euclidean distance and average linkage. The dendrogram scale shows the cluster distance.

**
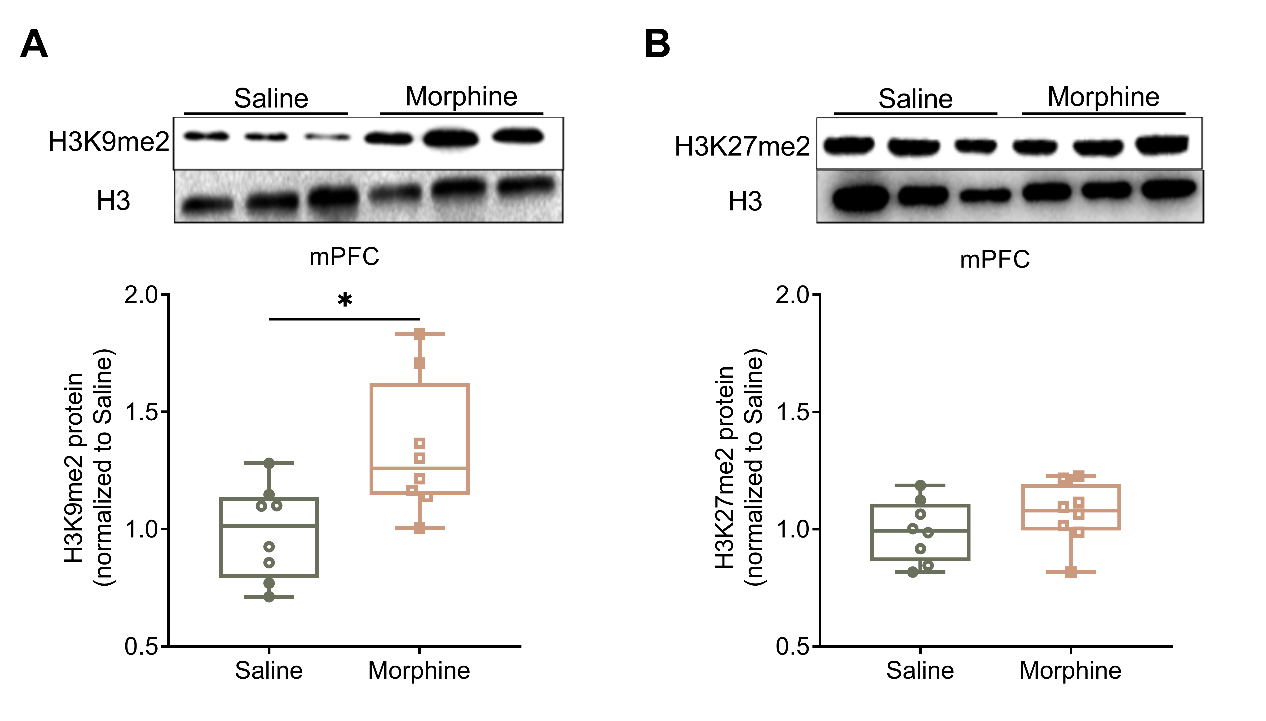
**

**Figure S10. Quantification of H3K9me2/H3K27me2 protein on Day 7 of withdrawal.** A) Changes in H3K9me2 protein on Day 7 of withdrawal (n = 8). The data were presented as the mean ± SEM; Unpaired t-test, **P* < 0.05. B) Changes in H3K27me2 protein on Day 7 of withdrawal (n = 8). The data were presented as the mean ± SEM; Unpaired t-test.

**
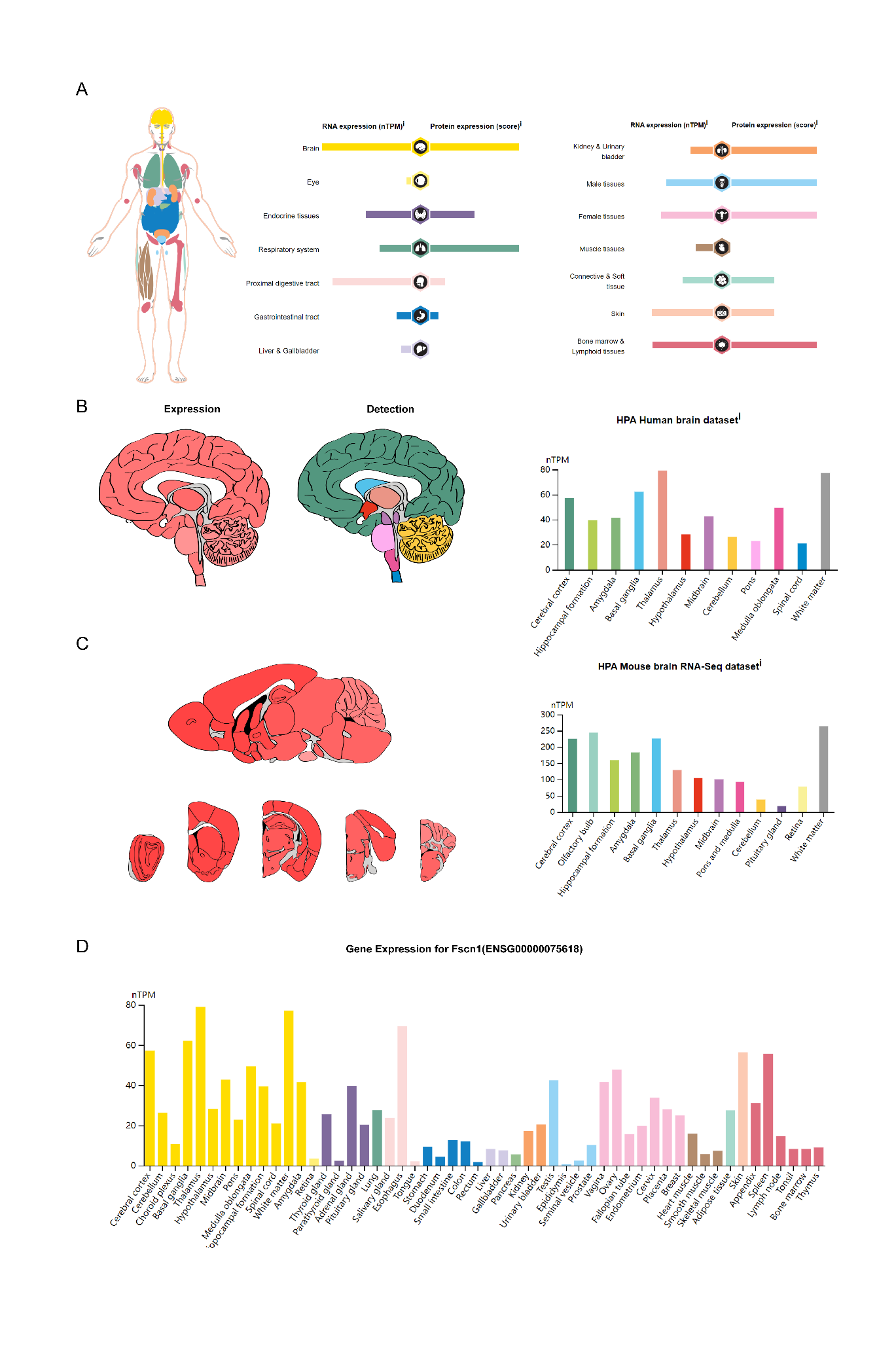
**

**Figure S11. The mRNA and protein expression of Fscn1 in the human and mouse central nervous system.** A) Fscn1 mRNA and protein expression data generated in the Human Protein Atlas project. B) HPA Human brain dataset generated in the Human Protein Atlas project. C) HPA Mouse brain RNA-Seq dataset generated in the Human Protein Atlas project. D) Gene expression for Fscn1 (ENSG00000075618) generated in the Human Protein Atlas project.
